# Does mobile phone survey method matter? Reliability of computer-assisted telephone interviews and interactive voice response non-communicable diseases risk factor surveys in low and middle income countries

**DOI:** 10.1371/journal.pone.0214450

**Published:** 2019-04-10

**Authors:** George W. Pariyo, Abigail R. Greenleaf, Dustin G. Gibson, Joseph Ali, Hannah Selig, Alain B. Labrique, Gulam Muhammed Al Kibria, Iqbal Ansary Khan, Honorati Masanja, Meerjady Sabrina Flora, Saifuddin Ahmed, Adnan A. Hyder

**Affiliations:** 1 Department of International Health, Johns Hopkins Bloomberg School of Public Health, Baltimore, MD, United States of America; 2 Department of Population, Family and Reproductive Health, Johns Hopkins Bloomberg School of Public Health, Baltimore, MD, United States of America; 3 Berman Institute of Bioethics, Johns Hopkins University, Baltimore, MD, United States of America; 4 Institute of Epidemiology, Disease Control and Research, Dhaka, Bangladesh; 5 Ifakara Health Institute, Dar es Salaam, United Republic of Tanzania; 6 George Washington University Milken Institute School of Public Health, Washington DC, United States of America; George Institute for Global Health, INDIA

## Abstract

**Introduction:**

Increased mobile phone subscribership in low- and middle-income countries (LMICs) provides novel opportunities to track population health. The objective of this study was to examine reliability of data in comparing participant responses collected using two mobile phone survey (MPS) delivery modalities, computer assisted telephone interviews (CATI) and interactive voice response (IVR) in Bangladesh (BGD) and Tanzania (TZA).

**Methods:**

Using a cross-over design, we used random digit dialing (RDD) to call randomly generated mobile phone numbers and recruit survey participants to receive either a CATI or IVR survey on non-communicable disease (NCD) risk factors, followed 7 days later by the survey mode not received during first contact; either IVR or CATI. Respondents who received the first survey were designated as first contact (FC) and those who consented to being called a second time and subsequently answered the call were designated as follow-up (FU). We used the same questionnaire for both contacts, with response options modified to suit the delivery mode. Reliability of responses was analyzed using the Cohen’s kappa statistic for percent agreement between two modes.

**Results:**

Self-reported data on demographic characteristics and NCD behavioral risk factors were collected from 482 (CATI-FC) and 653 (IVR-FC) age-eligible and consenting respondents in BGD, and from 387 (CATI-FC) and 674 (IVR-FC) respondents in TZA respectively. Survey follow-up rates were 30.7% (n = 482) for IVR-FU and 53.8% (n = 653) for CATI-FU in BGD; and 42.4% (n = 387) for IVR-FU and 49.9% (n = 674) for CATI-FU in TZA respectively. Overall, there was high consistency between delivery modalities for alcohol consumption in the past 30 days in both countries (kappa = 0.64 for CATI→IVR (BGD), kappa = 0.54 for IVR→CATI (BGD); kappa = 0.66 for CATI→IVR (TZA), kappa = 0.76 for IVR→CATI (TZA)), and current smoking (kappa = 0.68 for CATI→IVR (BGD), kappa = 0.69 for IVR→CATI (BGD); kappa = 0.39 for CATI→IVR (TZA), kappa = 0.50 for IVR→CATI (TZA)). There was moderate to substantial consistency in both countries for history of checking for hypertension and diabetes with kappa statistics ranging from 0.43 to 0.67. There was generally lower consistency in both countries for physical activity (vigorous and moderate) with kappa statistics ranging from 0.10 to 0.41, weekly fruit and vegetable with kappa ranging from 0.08 to 0.45, consumption of foods high in salt and efforts to limit salt with kappa generally below 0.3.

**Conclusions:**

The study found that when respondents are re-interviewed, the reliability of answers to most demographic and NCD variables is similar whether starting with CATI or IVR. The study underscores the need for caution when selecting questions for mobile phone surveys. Careful design can help ensure clarity of questions to minimize cognitive burden for respondents, many of whom may not have prior experience in taking automated surveys. Further research should explore possible differences and determinants of survey reliability between delivery modes and ideally compare both IVR and CATI surveys to in-person face-to-face interviews. In addition, research is needed to better understand factors that influence survey cooperation, completion, refusal and attrition rates across populations and contexts.

## Introduction

The International Telecommunications Union predicts that by 2020, the number of mobile phone subscriptions in sub-Saharan Africa (SSA) will be equal to the region’s population [1]. In South Asia, half of the population already owns a phone, and penetration levels will surpass 60% by 2025 [2]. Increased mobile phone ownership offers positive benefits for a number of sectors, including education, banking, and public health. In SSA, public health practitioners have used mobile phones for conducting surveillance [3,4] and emergency response [5,6]. Others have used them for cross-sectional [7,8] and panel surveys [9], behavior change communication, monitoring and evaluation, and training of health care providers [10,11].

Concomitant to the increase in cell phone ownership is a desire for more timely public health data for decision making. Non-communicable diseases (NCDs) are receiving additional public health attention in low- and middle-income countries (LMICs), as NCD prevalence grows [12,13]. The Sustainable Development Goals (SDG) call for annual monitoring of indicators. The third goal (SDG 3), “Ensure Healthy Lives and Promote Well-Being for All at All Ages”, calls for a one-third reduction of premature mortality from NCDs by the year 2030 [14]. The potential for mobile phone surveys (MPS) to improve public health response to NCDs has been previously discussed [12]. What is unknown, however, is the MPS data collection method in LMICs that would yield the most reliable results.

There are three main modes of mobile phone-based data collection: computer-assisted telephone interviews (CATI), interactive voice response (IVR) and short message service (SMS) [4,15–18]. CATI uses a live interviewer, usually in a call center, who interacts with the respondent by using a computer or tablet to read questions and record data [4,15]. In IVR, respondents press the digit on their numeric keypad that corresponds to a set of answers provided in a pre-recorded message that automatically plays [18,19]. In SMS surveys, researchers use text messages to communicate between electronic devices.

The mode of data collection (e.g. CATI, IVR, in-person) impacts survey participation and responses; response and measurement effects introduced by a mode are jointly referred to as mode effects [20]. Understanding mode effects is important because the mode used can introduce three types of errors: a) frame error, in which certain members of the target population are erroneously included or excluded from the sample frame; b) non-response error, when those who respond to the survey are different from those who do not respond to the survey; c) measurement error, due to the responses recorded in the survey not being accurate either due to respondent or interviewer error [21]–this study directly addresses the later.

Frequently documented in high income settings, there is a paucity of published literature comparing quality of data and reliability of MPS modes in LMICs [22] and few publications on conducting population-based MPS in LMIC settings [4]. The lack of knowledge about the reliability of results collected via different MPS modes (CATI, IVR or SMS) limits uptake of these approaches in LMICs. Increasing mobile penetration across populations suggests that these approaches may be an under-utilized resource to obtain population-level data. MPS may potentially be less costly and easier to implement than in-person sample surveys. The primary aim of the present study was to contribute to filling the gap in empirical evidence on mode effects in LMICs by evaluating and documenting the consistency of responses between NCD behavioral risk factor data collected using CATI and IVR in Bangladesh and Tanzania.

## Literature review and theoretical framework

Our team has previously conducted an extensive review of the literature in which we found very little published on population based mobile phone surveys in LMIC settings [4]. Of the more than 6,625 articles reviewed, only 11 articles contained information on MPS, and the majority of these were panel surveys. Most studies were conducted using CATI and the available evidence did not allow for meaningful analysis of mode effects in LMICs. There is very little that has been documented on mode effects in LMICs. Another literature review by our team focused on reliability and accuracy across mobile phone survey modalities in LMICs. Again, we found very few empirical studies reporting on mode effects, and even when reported, the heterogeneity of outcomes and limited number of comparisons of different modes precluded drawing any significant conclusions on mode effects in LMICs [22].

Drawing from the available literature on telephone survey methods and mode effects, we highlight some of the theoretical and conceptual issues that would be expected to impact the quality of data collected using two different modes of mobile phone survey delivery in LMICs. There are three common sources of error in mobile phone surveys. These are; a) frame error, in which certain members of the target population are erroneously included or excluded from the sample frame i.e., not every individual has a non-zero probability of being included in the survey sample; b) non-response error, when those who respond to the survey are different from those who do not respond to the survey; c) measurement error, due to the responses recorded in the survey not being accurate, either due to respondent or interviewer error [21]. In the context of MPS, frame error is concerning in countries where cell phone ownership is not above 80%, as not having a cell phone *a priori* excludes certain individuals [23]. Non-response error arises when sampled individuals do not respond or are under-represented for various reasons and are different from respondents.

Measurement error, which is the error most relevant to this study, can arise in three forms. The first source of measurement error is when a respondent intentionally misleads the interviewer or records responses which the respondent considers to be more socially acceptable. This phenomenon is referred to as social desirability bias and has been documented by survey researchers [24–27]. Tourangeau and Yan (2007) concluded that misreporting due to social desirability bias can be quite common with an interviewer administered survey especially for sensitive questions [25]. This may happen because respondents seek to present themselves in what they consider to be a more socially favorable light. This dynamic is more evident in interviewer administered, in-person surveys. In a wide ranging review of studies, the Pew Research Center has also concluded that interviewer administered telephone interviews (such as CATI) are more likely to report more socially desirable responses than self-administered surveys such as web surveys [27]. The limited evidence published on studies in LMICs is inconclusive, and what exists does not examine any MPS. The second scenario for measurement error is when the respondent un-intentionally provides an answer that is not reflective of her or his true views or experiences. Social researchers using telephone surveys have noted that the pressure to provide a response may lead to errors due to limited recall time even in interviewer administered surveys [27]. To limit the amount of effort in recalling and reporting an answer, a respondent may provide the first response option which comes to mind; a phenomenon referred to as satisficing and originally described by Herbert Simon in 1945 [28]. Another situation leading to measurement error is when the respondent agrees with a response option provided by the interviewer, even if it may not be reflective of her or his situation, a phenomenon referred to as acquiescence [24,29]. A third source of measurement error may be attributed to an interviewer not recording accurately the response provided by a research subject. Ongena and Djikstra (2007) argue that the presence of an interviewer in a CATI survey may introduce measurement error due to cognitive processes at play in the interaction between the interviewer and respondent. Such interviewer introduced measurement error would be present in a CATI survey and not in an IVR. One advantage of CATI over IVR is that an interviewer can keep the respondent engaged in the survey and provide clarifications due to a natural tendency to comply with rules of conversation [29], and this might be expected to increase the chances of a respondent providing more accurate responses, than in an automated survey such as IVR. However, the rules of conversation also mean that CATI interviewers are at risk of deviating from the text of the survey question, whereas an IVR survey delivers the question in exactly the same way to all participants who respond to it. Centralized supervision of interviewers in CATI surveys may help to reduce variation due to interviewer adaptation [30].

It has been reported that telephone respondents in general are more likely to complain about the length of surveys than face-to-face (F2F) surveys due to the social distance and the different dynamics at play in a F2F survey compared to a remotely administered survey [31]. Following the rules of conversation, we may expect a CATI respondent to be under more pressure to provide a reasonable response, one which makes sense to an interviewer, and thus less likely to satisfice than in an IVR survey. The social pressure to provide a reasonable response which occurs in an interviewer administered survey is less at play when a respondent is responding to an automated survey. However, the pressure to provide a quick response is more acutely felt by a respondent in an IVR survey than in a CATI survey. The cognitive burden involved in responding to a remotely administered survey is higher in IVR compared to CATI. The higher the cognitive burden in a survey, the more likely that a respondent will adapt a strategy, such as satisficing, to finish the survey as quickly as possible [32]. All these reasons could introduce differences in responses provided by the same respondent to the same set of questions delivered using CATI and IVR.

In combining modes or comparing results from surveys collected using different modes, researchers should be concerned and should attempt to design surveys and the questions in such a way as to reduce each of the three sources of error.

**Fig 1:**
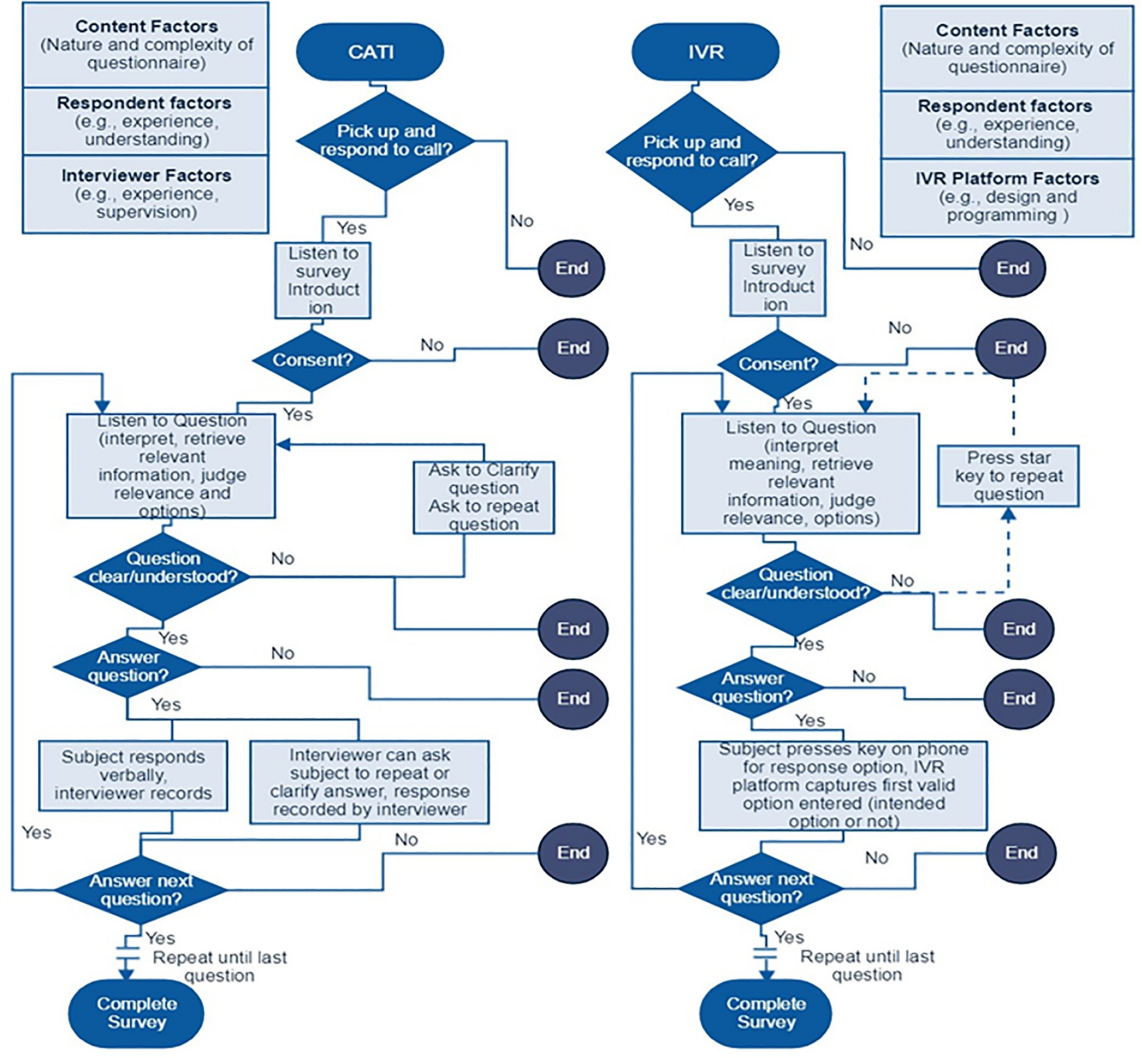
Conceptual framework showing key cognitive and decision pathways that influence accuracy of responses in computer assisted telephone interviews and interactive voice response surveys. CATI–Computer Assisted Telephone Interviews; IVR–Interactive Voice Response.

Fig 1 summarizes the key cognitive and decision pathways that a respondent would probably undertake in a mobile phone survey using CATI or IVR (Fig 1). Survey researchers generally agree that there are four key cognitive actions a survey respondent undertakes in answering a question in a survey; a) understand the question, b) retrieve relevant information from memory, c) decide on relevant information and accuracy with regard to the specific question, and d) formulate and provide a response to the question [29,33,34]. In order to answer a survey question, a respondent should be able to listen and understand the purpose of the survey, accept to participate, listen to and understand the question, reflect on her or his own experience, and provide an appropriate response. This process is repeated for all the questions in the survey. One might therefore expect that any difficulty along this cognitive and decision pathway could lead to a respondent to either provide a response or not, and when a response is provided, it may be recorded accurately and reflect the respondents true experience or views or it may be inaccurately recorded. This chain of events involves a respondent processing the words in a survey, choosing and providing a response to an interviewer (CATI) or pressing a telephone key pad corresponding to the appropriate intended responses (IVR).

One might expect that a complicated question, for example one that is long, has many words, many response options, or is on a non-familiar subject, may be harder for a respondent to provide an accurate response [33]. For surveys collecting information from a random sample of respondents using two different delivery modes, the comparability of responses may suffer from two main sources of error–non-response and measurement errors. Some respondents may provide responses to certain questions in one survey and not in the other resulting in non-response error. Second, errors may arise in the recording of a respondent’s answers and the answers may not reflect her or his intended response resulting in measurement error. Therefore, one might expect measurement errors even in an interviewer administered survey such as CATI, where a respondent has a chance to ask for clarifications, and interviewer can prompt for understanding of the question or repeating a response before recording a response. This is not possible for automated surveys such as IVR, where there is no possibility to ask for clarifications or to correct a response that has already been provided. The dynamics at play in CATI and IVR could result in discrepancy between answers provided by the respondent to the same survey using the two delivery modes.

## Materials and methods

### Study setting

We collected data in Bangladesh in partnership with the Institute of Epidemiology, Disease Control and Research (IEDCR) and in Tanzania with Ifakara Health Institute (IHI). Ethical approvals were obtained from the institutional review boards at the Johns Hopkins Bloomberg School of Public Health (JHSPH), IEDCR, and in Tanzania, both IHI and the National Institute of Medical Research.

In Bangladesh, the IEDCR conducts on-going MPS using CATI for surveillance of NCD risk factors and already had locally developed/adapted CATI software and a team of interviewers experienced in conducting such surveys. In Tanzania, the IHI collaborated with a local data technology firm which developed CATI software and used a team of interviewers experienced in conducting social sector and commercial surveys but, unlike Bangladesh, not NCD surveys. In both countries the research team trained interviewers on the specific NCD risk factor questionnaire used in the survey, interviewing techniques, and ethical data collection and protection.

### Study design

We used a random digit dialing (RDD) approach [35] to generate pools of potential phone numbers. All of the mobile network operators (MNOs) registered and active in the country and their unique prefixes that lead the 10-digit mobile phone number were identified. Using these unique prefixes, the remaining digits were randomly generated via a computer to create pools of mobile phone numbers to which the first contact surveys were to be delivered [23]. In both countries, we chose starting pools for IVR to be bigger than CATI starting pools because we had anticipated, based on initial tests of the IVR platform, that IVR may need many more calls to get a completed response than CATI, although the extent of this difference was unknown.

**Fig 2:**
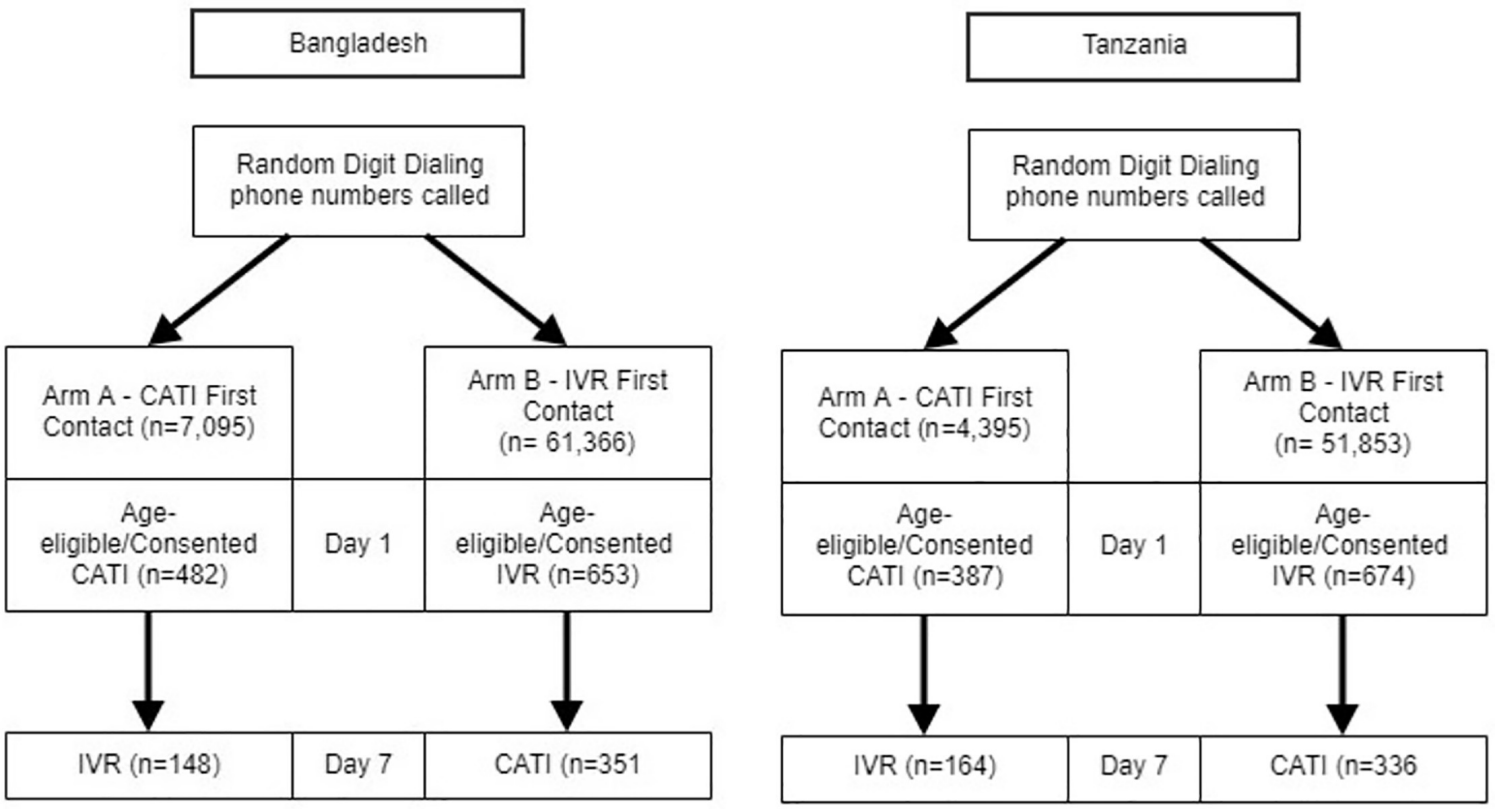
Cross-over design and samples for computer assisted telephone interviews and interactive voice response mobile phone surveys in Bangladesh and Tanzania. CATI–Computer Assisted Telephone Interviews; IVR–Interactive Voice Response.

In each country, using a cross-over design, the aforementioned pools of randomly generated mobile phone numbers were employed to start each of the two study arms. Respondents who received the first survey of either modality were designated as first contact (FC) and those who consented to being called a second time and subsequently answered the call were designated as follow up (FU). Participants in the first study arm, (CATI→IVR), received a CATI survey on first contact (CATI-FC), followed by an IVR survey seven days later (IVR-FU). Participants in the second study arm, (IVR→CATI) received an IVR survey on first contact (IVR-FC) followed by a CATI survey seven days later (CATI-FU) (Fig 2). If a respondent answered the call, he or she was asked to provide age and consent. Participants who indicated an age less than 18 years old were excluded from the study. Those who were age-eligible but refused to consent were not interviewed.

At first contact, the interviewer in the initial CATI arm (CATI-FC) or the automated recording in the initial IVR arm (IVR-FC) informed the participants that they would be contacted a second time after one week. The questions used in both study arms were the same, albeit with response options adjusted to mode. In the IVR survey, if the respondent did not provide a response within the valid range of answers within 7 seconds, the question would repeat up to three times.

The choice of the crossover design allowed for assessment of response consistency, adjusted for the risk of “priming” after exposure to the prior modality [36–38]. During the information and consent process, all respondents were informed of an airtime incentive, to be delivered as a credit to their mobile phone account on completion of the survey. The amount, timing, and structure of incentive provided was based on information obtained during the formative phase. Additional details on study methods employed across all study sites are contained in our research protocol which was previously published elsewhere [39].

### Questionnaire

An expert group drawn from JHSPH, Centers for Disease Control and Prevention (CDC), the World Health Organization (WHO) and University of Buffalo developed the questionnaire used in the study. The group adapted questions used previously in other established surveys including WHO’s Stepwise Approach to Surveillance of NCD risk factors (STEPs) surveys [40,41], tobacco questions for surveys [42] and CDC’s Behavioral Risk Factor Surveillance System [39,43]. To ensure the questionnaires were appropriate for local contexts, we conducted formative research in consultation with in-country experts [39]. Translations and back-translation into the one main national language in each country, i.e., Bangla in Bangladesh and Kiswahili in Tanzania, helped identify locally appropriate examples. The questionnaire had 6 modules covering demographics, tobacco (smoked and smokeless), alcohol, diet (fruit, vegetables and salt), physical activity (vigorous and moderate), and medical conditions (blood pressure and diabetes). The questionnaires used in both countries are available (S1 File and S2 File).

### Data collection

After the introductory message and demographic questions, the IVR platform then randomly ordered the six aforementioned blocks of domain-specific NCD questions to determine the sequence of question delivery in both arms. This was to control for effects of likely time-related survey drop-off and respondent fatigue different from that due to the survey content itself [44,45]. The order of individual questions in each domain module remained the same to preserve skip patterns.

The IVR surveys and CATI survey calls were placed between 8:00 am—8:00 pm local time in both Bangladesh and Tanzania. If the targeted respondent missed the initial call, the IVR platform or CATI enumerator made three additional attempts to the same number. Calls were made available in Bangla and English in Bangladesh, and in Kiswahili and English in Tanzania, and respondents could select the preferred survey language by pressing a number on their telephone keypad for IVR or telling the enumerator at the start of CATI surveys.

Data were collected from late-June to mid-July 2017 in Tanzania and mid-August to late-August 2017 in Bangladesh.

### Analysis

Data were extracted from the IVR platform in comma separated values (CSV) format [46]. CATI data were collected using Open Data Kit software and were also downloaded from a cloud server in CSV format. Both CATI and IVR data were imported, cleaned and analyzed using Stata Version 14 [47]. We focused the analysis on NCD risk factors similar to the types of indicators reported in WHO recommended NCD behavioral risk factor surveys such as STEPs and which countries are encouraged to monitor and report regularly [40,41]. If a respondent is administered the same survey within a short recall period, such as one week, in order to minimize recall bias, one might expect that if the two delivery modalities are comparable, the respondent should return the same responses to identical questions. An NCD question to which a respondent provided an answer in both first contact and follow up survey was included in the analysis of reliability between CATI and IVR delivery modes. We compared each respondent’s own answers to the same question between the two MPS modes variable by variable for selected key demographic and NCD behavioral questions. Respondents who answered the question in both first contact and follow-up surveys were included in the analysis of reliability between delivery modes. Consistency of responses between the two delivery modalities was analyzed using Cohen’s Kappa statistic for percent agreement among two raters [48–50]. The Kappa statistic is a measure ranging from -1 to +1, with the extremes representing perfect disagreement and perfect agreement respectively, and 0 representing an observed agreement one would get purely by chance [50,51]. Landis and Koch (1977) suggested categorization and interpretation of intermediate values of Kappa as being: below 0.0 (Poor); 0.00–0.20 (Slight); 0.21–0.40 (Fair); 0.41–0.60 (Moderate); and 0.61–0.80 (Substantial) [51]. Since the kappa statistic is computed among those who answered both surveys, participants who only answered the first survey and not the second were excluded.

We performed sensitivity analyses by calculating the kappa statistic for different scenarios including; a) after dropping incomplete data, b) keeping only those who had complete demographic data and had answered at least one of the NCD modules, and, c) keeping only those who provided the exact same response in both surveys to demographic questions of age, gender and schooling. Furthermore, we explored associations between the length of the question, proxied by the number of words and audio duration of question in the local language, and the consistency of responses between the two modes (measured by the corresponding kappa statistic for each variable), using the Pearson product moment correlation coefficient and the associated degrees of freedom (df) and p-values for test of significance.

### Sample and survey participation

First contact (FC) self-reported data on demographic characteristics and NCD behavioral risk factors were collected from up to 482 (CATI-FC) and 653 (IVR-FC) age-eligible and consenting respondents in Bangladesh (BGD), and from up to 387 (CATI-FC) and 674 (IVR-FC) similar respondents in Tanzania (TZA) respectively (Table 1). Survey follow-up rates were 30.7% (n = 482) for IVR-FU (BGD), 53.8% (n = 653) for CATI-FU (BGD) and 42.4% (n = 387) for IVR-FU (TZA) and 49.9% (n = 674) for CATI-FU (TZA). Respondents were predominantly male across both survey delivery modalities in both countries. Overall median age was similar across survey arms and delivery modalities with a majority of respondents in the younger age-group of 18–29.

**Table 1:** Demographic characteristics of survey respondents by arm in Bangladesh and Tanzania mobile phone surveys.

	Bangladesh				Tanzania			
	Arm 1		Arm 2		Arm 1		Arm 2	
	CATI* First Contact (CATI-FC)	IVR** Follow up (IVR-FU)	IVR First Contact (IVR-FC)	CATI Follow up (CATI-FU)	CATI First Contact (CATI-FC)	IVR Follow up (IVR-FU)	IVR First Contact (IVR-FC)	CATI Follow up (CATI-FU)
**Reported Age, n (= > 18 years)**	482 (100%)	148 (100%)	653 (100%)	351 (100%)	387 (100%)	164 (100%)	674 (100%)	336 (100%)
**Age—median (IQR)**	27 (22, 35)	27 (20, 35)	25 (20, 32)	24 (20, 28)	32 (25, 42)	28 (22, 36)	26 (22, 32)	27 (22, 33)
**Age-group**								
**18–29**	277 (57%)	84 (57%)	448 (69%)	274 (78%)	164 (42%)	95 (58%)	442 (66%)	210 (63%)
**30–49**	174 (36%)	51 (34%)	137 (21%)	67 (19%)	172 (44%)	61 (37%)	192 (28%)	115 (34%)
**50–69**	31 (6%)	6 (4%)	28 (4%)	10 (3%)	42 (11%)	7 (4%)	27 (4%)	9 (3%)
**18–69**	**482 (100%)**	**141 (95%)**	**613 (94%)**	**351 (100%)**	**378 (98%)**	**163** **(99%)**	**661** **(98%)**	**334** **(99%)**
**70+**	-	7 (5%)	40 (6%)	-	9 (2%)	1 (1%)	13 (2%)	2 (1%)
**Sex**								
**Female**	170 (35%)	42 (28%)	81 (12%)	35 (10%)	143 (37%)	45 (27%)	178 (26%)	89 (26%)
**Male**	311 (65%)	106 (72%)	555 (85%)	316 (90%)	244 (63%)	119 (73%)	494 (73%)	247 (74%)
**Location**								
**Rural**	287 (60%)	83 (56%)	254 (39%)	142 (40%)	132 (34%)	71 (43%)	274 (41%)	119 (35%)
**Urban**	192 (40%)	64 (43%)	372 (57%)	209 (60%)	256 (66%)	106 (65%)	385 (57%)	217 (65%)
**Highest education level attempted**								
**No school**	64 (13%)	7 (5%)	59 (9%)	34 (10%)	22 (6%)	9 (5%)	50 (7%)	7 (2%)
**Primary**	182 (38%)	33 (22%)	133 (20%)	71 (20%)	217 (56%)	80 (49%)	306 (45%)	155 (46%)
**Secondary**	82 (17%)	48 (32%)	187 (29%)	82 (23%)	118 (30%)	56 (34%)	255 (38%)	136 (40%)
**University/tertiary plus**	148 (31%)	59 (40%)	239 (37%)	162 (46%)	28 (7%)	14 (9%)	7 (1%)	38 (11%)

NB:

*CATI–Computer Assisted Telephone Interviews

**IVR–Interactive Voice Response; FC–First Contact; FU–Follow Up

Key: CATI-FC–Arm 1, CATI delivered as first contact; IVR-FU–Arm 1, IVR as follow up; IVR-FC–Arm 2, IVR delivered as first contact; CATI-FU–Arm 2, CATI as follow up, IQR- Inter-quartile range

There was varied participation for different demographic and NCD variables. Of all age-eligible and consenting first contact respondents (n = 482) in Bangladesh, 176 respondents completed all questions in the demographic module in both CATI-FC (36.5%, n = 482) and IVR-FU surveys (94.1%, n = 187); while 340 respondents completed all demographic questions in both IVR-FC (52.1%, n = 653) and CATI-FU surveys (96.9%, n = 351). In Tanzania, 167 CATI-FC respondents answered all demographic questions (43.1%, n = 387) and IVR-FU (97.1%, n = 172) and 335 respondents for IVR-FC (49.7%, n = 674) and CATI-FU (99.7%, n = 336) respectively. Some respondents who had initially consented and started the survey subsequently withdrew or simply hung-up.

Most of the respondents who answered the demographic questions went on to answer at least one NCD question. Considering those who answered all demographic questions and at least one NCD question; in Bangladesh 170 respondents answered in both CATI-FC (35.3%, n = 482) and IVR-FU (90.9%, n = 187) surveys; while 340 respondents answered all demographic questions and at least one NCD question in both IVR-FC (52.1%, n = 653) and CATI-FU (96.9%, n = 351) surveys. In Tanzania the corresponding figures answering all demographic questions and at least one NCD question were 164 respondents for CATI-FC (42.4%, n = 387) and IVR-FU (95.3%, n = 172) and 332 respondents for IVR-FC (49.3%, n = 674) and CATI-FU (98.8%, n = 336) respectively.

Survey response rates were calculated according to definitions of the American Association for Public Opinion Research (AAPOR) [52] and are shown in Table 2.

**Table 2:** Survey response rates by study arm and mobile phone delivery mode in Bangladesh and Tanzania.

	Bangladesh				Tanzania			
	Arm 1		Arm 2		Arm 1		Arm 2	
AAPOR Category	CATI* First Contact (CATI-FC)	IVR** Follow up (IVR-FU)	IVR First Contact (IVR-FC)	CATI Follow up (CATI-FU)	CATI First Contact (CATI-FC)	IVR Follow up (IVR-FU)	IVR First Contact (IVR-FC)	CATI Follow up (CATI-FU)
**Contact Rate #1**	1,005/7,059 (14.2%)	163/338 (48.2%)	829/61,129 (1.4%)	328/403 (81.4)	455/4,391 (10.4%)	137/379 (36.1%)	730/51,759 (1.4%)	356/396 (89.9%)
**Response Rate #2**	429/7,059 (6.1%)	88/338 (26.0%)	558/61,129 (0.9%)	319/403 (79.2%)	384/4,391 (8.7%)	91/379 (24.0%)	557/51,759 (1.1%)	335/396 (84.6%)
**Refusal Rate #1**	576/7,059 (8.2%)	75/338 (22.2%)	271/61,129 (0.4%)	9/403 (2.2%)	71/4,391 (1.6%)	46/379 (12.1%)	173/51,759 (0.3%)	21/396 (5.3%)
**Cooperation Rate #2**	429/1,005 (42.7%)	88/163 (54.0%)	558/829 (67.3%)	319/328 (97.3%)	384/455 (84.4%)	91/137 (66.4%)	557/730 (76.3%)	335/356 (94.1%)

NB:

*CATI–Computer Assisted Telephone Interviews

**IVR–Interactive Voice Response; FC–First Contact; FU–Follow Up

Key: CATI-FC–Arm 1, CATI delivered as first contact; IVR-FU–Arm 1, IVR as follow up; IVR-FC–Arm 2, IVR delivered as first contact; CATI-FU–Arm 2, CATI as follow up

The AAPOR equations used in calculating the survey response rates shown in Table 2 are available (S1 Table).

## Results

### Measurement effects

#### Demographic variables

**Fig 3:**
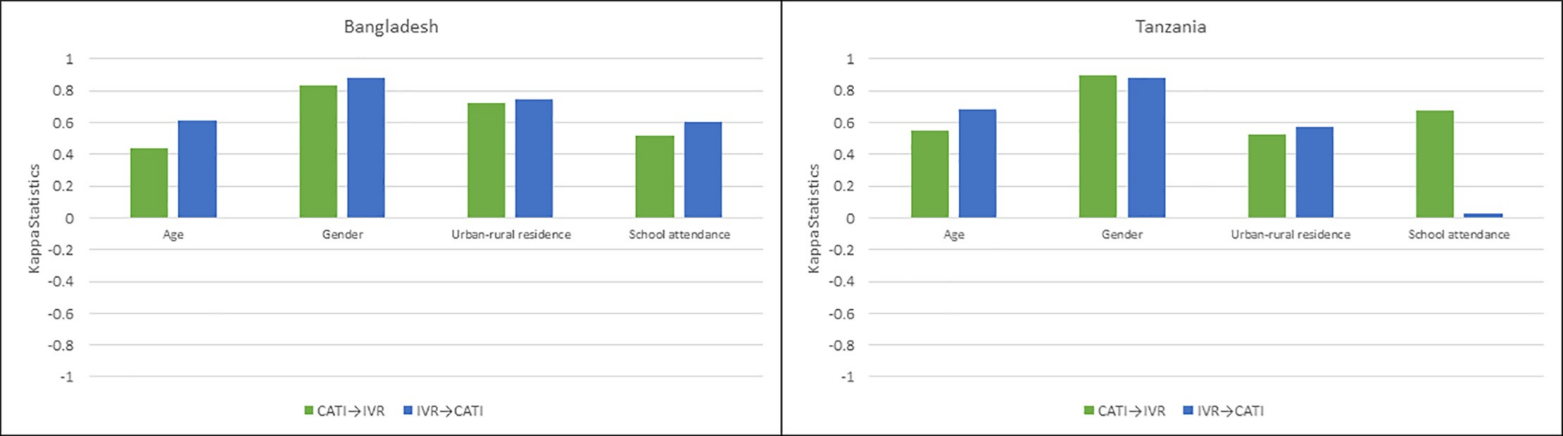
Kappa statistics comparing selected demographics in surveys using computer assisted telephone interviews and interactive voice response in Bangladesh and Tanzania. CATI–Computer Assisted Telephone Interviews; IVR–Interactive Voice Response; CATI**→**IVR indicates IVR as follow up mode (after CATI first contact). IVR**→**CATI indicates CATI as follow up mode (after IVR first contact).

We found moderate to substantial agreement for demographics between CATI and IVR in both Bangladesh and Tanzania regardless of which mode came first (Fig 3).

Among all variables, gender was the most consistently reported and had the highest kappa statistic in both countries (kappa = 0.83 for CATI→IVR (BGD), kappa = 0.88 for IVR→CATI (BGD); kappa = 0.88 for CATI→IVR (TZA), kappa = 0.88 for IVR→CATI (TZA)) as seen in Table 3.

**Table 3:** Percent agreement and kappa statistics in comparing selected demographics from computer assisted telephone interviews and interactive voice response mobile phone surveys in Bangladesh and Tanzania.

	Bangladesh		Tanzania	
	Arm 1	Arm 2	Arm 1	Arm 2
	IVR Follow up (CATI→IVR)	CATI Follow up (IVR→CATI)	IVR Follow up (CATI→IVR)	CATI Follow up (IVR→CATI)
**1. Age**	(n = 195)	(n = 342)	(n = 161)	(n = 335)
**Expected Agreement**	4.11%	5.87%	3.80%	4.26%
**Observed Agreement**	46.15%	63.16%	56.67%	69.55%
**Kappa statistic**	0.4385	0.6086	0.5496	**0.6820**
**S.E.**	0.0136	0.0132	0.0146	0.0114
**2. Gender**	(n = 180)	(n = 342)	(n = 173)	(n = 335)
**Expected Agreement**	57.35%	82.29%	60.69%	60.98%
**Observed Agreement**	92.78%	97.95%	95.95%	95.22%
**Kappa statistic**	**0.8307**	**0.8844**	**0.8971**	**0.8776**
**S.E.**	0.0736	0.0533	0.0760	0.0546
**3. Urban/rural residence**	(n = 178)	(n = 342)	(n = 171)	(n = 335)
**Expected Agreement**	51.40%	51.74%	53.10%	52.46%
**Observed Agreement**	86.52%	87.72%	77.78%	79.70%
**Kappa statistic**	**0.7226**	**0.7455**	0.5262	0.5730
**S.E.**	0.0749	0.0541	0.0763	0.0542
**4. Schooling**	(n = 179)	(n = 340)	(n = 168)	(n = 195)
**Expected Agreement**	21.85%	22.41%	38.08%	15.57%
**Observed Agreement**	62.01%	69.12%	79.76%	17.95%
**Kappa statistic**	0.5139	0.6020	**0.6732**	0.0282
**S.E.**	0.0385	0.0284	0.0554	0.0147

CATI–Computer Assisted Telephone Interviews; IVR–Interactive Voice Response; CATI→IVR indicates IVR as follow up mode (after CATI first contact). IVR→CATI indicates CATI as follow up mode (after IVR first contact).

NB: The kappa-statistic measure of agreement is scaled to be 0 when the amount of agreement is what would be expected to be observed by chance; -1 would represent perfect disagreement; and +1 would represent perfect agreement. For intermediate values, Landis and Koch (1977a, 165) suggest the following interpretations: below 0.0 Poor; 0.00–0.20 Slight; 0.21–0.40 Fair; 0.41–0.60 Moderate; **0.61–0.80 Substantial; 0.81–1.00 Almost perfect. Bolded kappa statistics in the table represent the ‘substantial’, and ‘almost perfect’ agreement categories.**

Age was reported as a continuous variable in the survey, although categorized at analysis into four age groups (18–29, 30–49, 50–69 and 70+). Reliability of reported urban or rural residence was higher in Bangladesh than Tanzania as evidenced by higher values of kappa statistics, and neither country showed a marked difference in reliability by mode order (kappa = 0.72 for CATI→IVR (BGD), kappa = 0.74 for IVR→CATI (BGD); kappa = 0.53 for CATI→IVR (TZA), kappa = 0.57 for IVR→CATI (TZA)). Schooling was more consistently reported among those who had CATI as first contact in Tanzania with higher kappa statistics (kappa = 0.67 for CATI→IVR (TZA)) than Bangladesh (kappa = 0.51 for CATI→IVR (BGD)).

We were interested to learn the extent to which respondent understanding of the survey may have influenced subsequent observed consistency between modes for NCD variables. To explore this, we examined percentages of respondents who **provided the same exact response in both surveys** to the demographic questions. This served as a proxy for respondent understanding of the survey. In Bangladesh, 30.3% (n = 195) of CATI first contact respondents provided the same **age** as in the follow up survey using IVR, and 45.3% (n = 342) of IVR first contact respondents gave the same age as in the follow up CATI survey. The corresponding figures for Tanzania were 45.3% (n = 161) and 6.0% (n = 335) for CATI first contact and IVR first contact respectively. For **gender,** 33.3% (n = 180) CATI first contacts and 45.3% (n = 342) IVR first contacts in Bangladesh provided matching responses. In Tanzania, corresponding figures were 42.2% (n = 173) and 6.0% (n = 335) for CATI and IVR first contacts respectively. Others in Bangladesh were; **rural/urban residence**– 29.2% (n = 178) for CATI first contact; and 40.4% (n = 342) for IVR first contact. Corresponding figures for matching rural/urban residence in Tanzania were; 32.7% (n = 171) and 3.6% (n = 335) for CATI and IVR first contacts respectively. For **schooling** 29.6% (n = 179) for CATI first contact in Bangladesh; 45.6% (n = 340) for IVR first contact; while Tanzania had 43.5% (n = 168) of CATI first contact respondents and 10.3% (n = 195) IVR first contact respondents respectively providing matching responses for schooling in the follow up surveys.

#### Behavior variables

**Fig 4:**
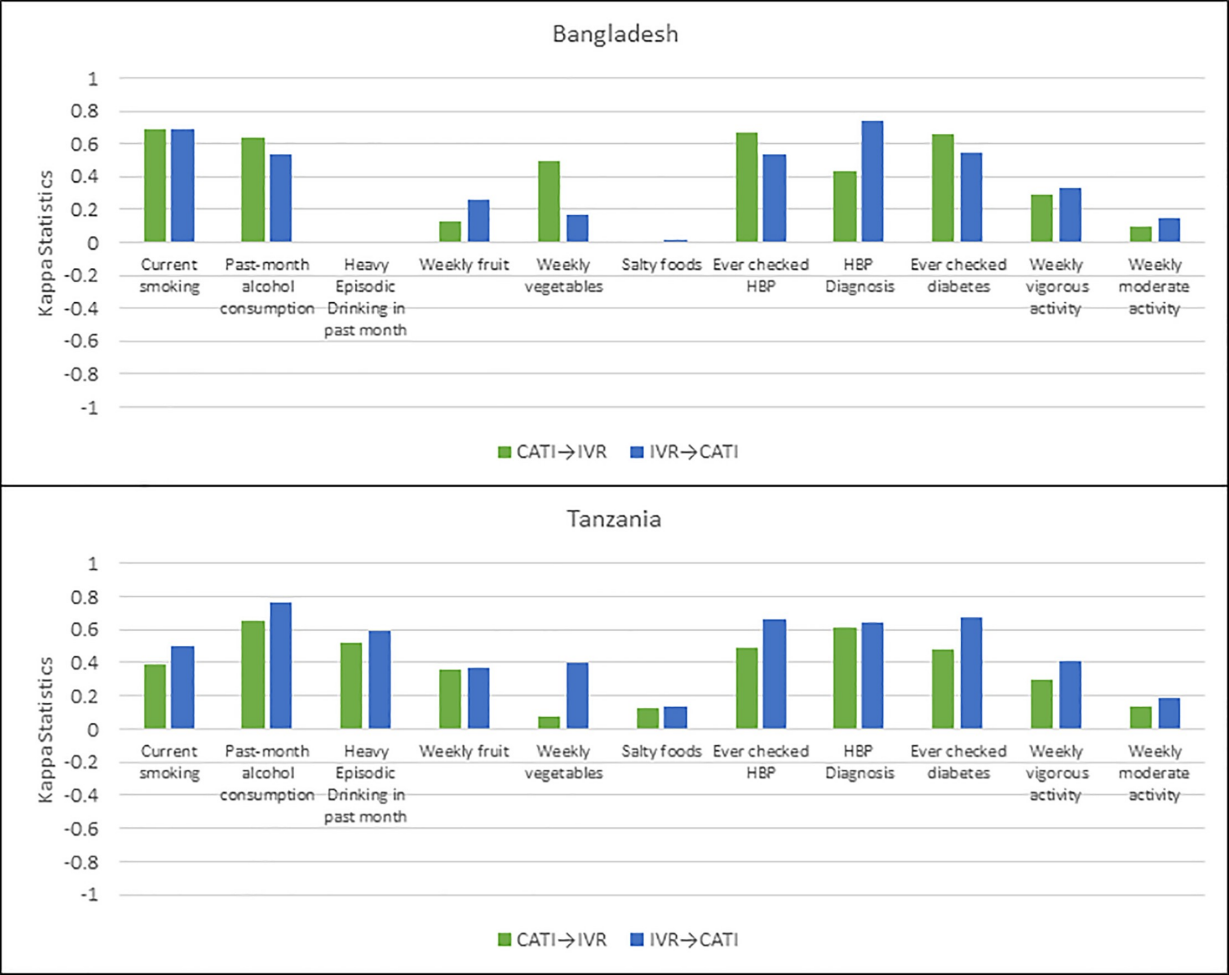
Kappa statistics comparing selected non-communicable disease risk factors in surveys using computer assisted telephone interviews and interactive voice response in Bangladesh and Tanzania. CATI–Computer Assisted Telephone Interviews; IVR–Interactive Voice Response; CATI**→**IVR indicates IVR as follow up mode (after CATI first contact). IVR**→**CATI indicates CATI as follow up mode (after IVR first contact).

Fig 4 shows the comparison of kappa statistics for consistency of responses on selected NCD behavioral risk factors between CATI and IVR in Bangladesh and Tanzania (Fig 4).

In general, similar to demographics, findings in Bangladesh and Tanzania generally followed the same pattern, albeit with a more mixed picture for NCD risk factors. Overall, there was high consistency between delivery modalities for alcohol consumption in the past 30 days in both countries (kappa = 0.64 for CATI→IVR (BGD), kappa = 0.54 for IVR→CATI (BGD); kappa = 0.66 for CATI→IVR (TZA), kappa = 0.76 for IVR→CATI (TZA)). Similarly, for current smoking (kappa = 0.68 for CATI→IVR (BGD), kappa = 0.69 for IVR→CATI (BGD); kappa = 0.39 for CATI→IVR (TZA), kappa = 0.50 for IVR→CATI (TZA)). There was a moderate to substantial consistency in both countries for history of checking for hypertension and diabetes with kappa statistics ranging from 0.43 to 0.67. There was generally lower consistency in both countries for physical activity (vigorous and moderate) with kappa ranging from 0.10 to 0.41, weekly fruit and vegetable with kappa ranging from 0.08 to 0.45 and consumption of foods high in salt or efforts to limit salt with kappa statistics generally below 0.3 (Table 4).

**Table 4:** Percent agreement and kappa statistics in comparing selected non-communicable disease risk factors from computer assisted telephone interviews and interactive voice response mobile phone surveys in Bangladesh and Tanzania.

	Bangladesh		Tanzania	
	Arm 1	Arm 2	Arm 1	Arm 2
	IVR Follow up (CATI→IVR)	CATI Follow up (IVR→CATI)	IVR Follow up (CATI→IVR)	CATI Follow up (IVR→CATI)
**1. Smoking tobacco Currently**	(n = 154)	(n = 326)	(n = 149)	(n = 318)
**Expected Agreement**	46.42%	42.54%	82.33%	82.92%
**Observed Agreement**	83.12%	82.21%	89.26%	91.51%
**Kappa statistic**	**0.6849**	**0.6904**	0.3925	0.5030
**S.E.**	0.0610	0.0416	0.0585	0.0416
**2. Positive history of alcohol consumption last 30 days**	(n = 18)	(n = 50)	(n = 41)	(n = 84)
**Expected Agreement**	84.57%	65.36%	50.21%	49.74%
**Observed Agreement**	94.44%	84.00%	82.93%	88.10%
**Kappa statistic**	**0.6400**	0.5381	**0.6571**	**0.7631**
**S.E.**	0.2199	0.1376	0.1545	0.1060
**3. Positive history of alcohol—6 or more drinks last 30 days**	(n = 0)	(n = 7)	(n = 19)	(n = 33)
**Expected Agreement**	-	51.02%	56.23%	47.93%
**Observed Agreement**	-	42.86%	78.95%	78.79%
**Kappa statistic**	-	-0.1667	0.5190	0.5926
**S.E.**	-	0.3780	0.2227	0.1590
**4. Any fruit in typical week**	(n = 162)	(n = 339)	(n = 162)	(n = 330)
**Expected Agreement**	87.31%	81.38%	81.70%	82.62%
**Observed Agreement**	88.89%	86.14%	88.27%	89.09%
**Kappa statistic**	0.1243	0.2552	0.3590	0.3724
**S.E.**	0.0771	0.0398	0.0785	0.0480
**5. Any vegetable in typical week**	(n = 154)	(n = 324)	(n = 161)	(n = 331)
**Expected Agreement**	97.43%	89.99%	89.90%	88.00%
**Observed Agreement**	98.70%	91.67%	90.68%	92.75%
**Kappa statistic**	0.4951	0.1678	0.0772	0.3958
**S.E.**	0.0696	0.0308	0.0711	0.0503
**6. Consumption of processed foods high in salt (always/sometimes)**	(n = 154)	(n = 338)	(n = 151)	(n = 329)
**Expected Agreement**	13.64%	11.77%	32.13%	32.86%
**Observed Agreement**	12.99%	12.72%	40.40%	41.95%
**Kappa statistic**	-0.0076	0.0108	0.1218	0.1353
**S.E.**	0.0216	0.0123	0.0477	0.0348
**7. Effort to limit salt in diet**	(n = 154)	(n = 337)	(n = 150)	(n = 328)
**Expected Agreement**	39.02%	47.57%	47.75%	48.13%
**Observed Agreement**	43.51%	60.53%	52.00%	59.45%
**Kappa statistic**	0.0736	0.2473	0.0813	0.2182
**S.E.**	0.0507	0.0490	0.0736	0.0512
**8. Ever checked high blood pressure**	(n = 158)	(n = 332)	(n = 151)	(n = 328)
**Expected Agreement**	50.77%	49.66%	51.45%	56.92%
**Observed Agreement**	83.54%	76.81%	75.50%	85.37%
**Kappa statistic**	**0.6657**	0.5393	0.4953	**0.6603**
**S.E.**	0.0789	0.0540	0.0805	0.0552
**9. History of diagnosis of high blood pressure/taking HT medications**	(n = 76)	(n = 123)	(n = 43)	(n = 79)
**Expected Agreement**	69.74%	68.59%	75.99%	64.69%
**Observed Agreement**	82.89%	91.87%	90.70%	87.34%
**Kappa statistic**	0.4348	**0.7412**	**0.6126**	**0.6416**
**S.E.**	0.1007	0.0902	0.1525	0.1113
**10. Ever checked diabetes**	(n = 161)	(n = 335)	(n = 153)	(n = 329)
**Expected Agreement**	58.92%	62.36%	59.86%	62.56%
**Observed Agreement**	85.71%	82.69%	79.08%	87.84%
**Kappa statistic**	**0.6523**	0.5400	0.4789	**0.6753**
**S.E.**	0.0788	0.0544	0.0793	0.0551
**11. History of diagnosis of diabetes/taking diabetes medications**	(n = 35)	(n = 55)	(n = 26)	(n = 62)
**Expected Agreement**	59.02%	65.62%	92.31%	83.71%
**Observed Agreement**	88.57%	92.73%	92.31%	88.71%
**Kappa statistic**	**0.7211**	**0.7885**	0.0000	0.3067
**S.E.**	0.1674	0.1318	0.0000	0.1213
**12. Any vigorous physical activity in typical week**	(n = 167)	(n = 338)	(n = 153)	(n = 329)
**Expected Agreement**	46.69%	48.42%	58.17%	60.77%
**Observed Agreement**	62.28%	65.68%	70.59%	76.90%
**Kappa statistic**	0.2923	0.3346	0.2968	0.4112
**S.E.**	0.0678	0.0506	0.0754	0.0550
**13. Any moderate physical activity in typical week**	(n = 161)	(n = 338)	(n = 153)	(n = 328)
**Expected Agreement**	38.56%	45.96%	70.41%	76.42%
**Observed Agreement**	44.72%	54.14%	74.51%	80.79%
**Kappa statistic**	0.1002	0.1515	0.1386	0.1853
**S.E.**	0.0495	0.0453	0.0799	0.0492

CATI–Computer Assisted Telephone Interviews; IVR–Interactive Voice Response; CATI→IVR indicates IVR as follow up mode (after CATI first contact). IVR→CATI indicates CATI as follow up mode (after IVR first contact).

NB: The kappa-statistic measure of agreement is scaled to be 0 when the amount of agreement is what would be expected to be observed by chance; -1 would represent perfect disagreement; and +1 would represent perfect agreement. For intermediate values, Landis and Koch (1977a, 165) suggest the following interpretations: below 0.0 Poor; 0.00–0.20 Slight; 0.21–0.40 Fair; 0.41–0.60 Moderate; **0.61–0.80 Substantial, 0.81–1.00 Almost perfect. Bolded kappa statistics in the table represent the ‘substantial’, and ‘almost perfect’ agreement categories.**

Restricting analysis only to those respondents who answered all demographic questions and at least one NCD question in both first contact and follow up survey modalities did not show much difference in kappa statistics. Similarly, restricting analysis to those with the same answer to demographic questions only slightly improved kappa for NCD risk factor variables, albeit not much different from the unrestricted analysis (S2 Table).

### Question duration for selected variables

In Bangladesh, the call duration of first contact IVR connections, including those which broke off without any responses, ranged from 0.002 to 22.101 minutes with a median of 0.710 minutes (inter-quartile range [IQR]: 0.435, 1.098) (S3 Table). The country adapted questionnaire contained questions with English word counts ranging from 12 to 74, with a median of 24 words (IQR: 19, 40) which were then translated into Bangla, the main national language. The English questions audio duration ranged from 0.117 to 0.667 minutes, with a median of 0.183 minutes (IQR: 0.150, 0.283). However, all CATI respondents (100%) and the vast majority of respondents in both IVR first contact and follow up surveys (about 98%) answered the survey in Bangla. The Bangla questions audio duration ranged from 0.150 to 0.717 minutes, with a median of 0.250 minutes (IQR: 0.167, 0.367).

For Tanzania the call duration of first contact IVR connections, again including break-off calls, ranged from 0.020 to 23.889 minutes with a median of 0.564 minutes (IQR 0.481, 0.971). The country adapted questionnaire contained questions with English word counts ranging from 15 to 46, with a median of 23 words (IQR 18, 38) and these were then translated into Kiswahili, the main national language used in Tanzania. The English questions audio duration ranged from 0.133 to 0.500 minutes, with a median of 0.267 minutes (IQR 0.217, 0.433). All CATI respondents (100%) and the vast majority of respondents in both IVR first contact and follow up surveys (about 98%) took the survey in Kiswahili. The Kiswahili question audio duration ranged from 0.167 to 0.650 minutes, with a median of 0.300 minutes (IQR 0.233, 0.433).

In both countries, there was a consistent negative correlation between audio duration and the corresponding kappa statistic for reliability between modes for all variable pairs but these associations were not statistically significant as assessed using Pearson’s product moment correlation coefficient (S3 Table).

The Pearson correlation coefficient, associated degrees of freedom (df = 2), and p-value testing the significance of the correlation of audio duration and the corresponding kappa statistics for consistency between CATI first contact with IVR follow up for demographics in Bangladesh was r(2) = -0.167, p > 0.10 (two tailed); for audio duration and IVR first contact with CATI follow up was r(2) = -0.389, p > 0.10 (two tailed). The coefficients and associated degrees of freedom (df = 11), and p-value for the correlation between audio duration and kappa statistics for NCD variables were r(11) = -0.155, p > 0.10 (two tailed) for CATI first contact/IVR follow up and r(11) = -0.090, p > 0.10 (two tailed).

Similarly, the correlation coefficient, associated degrees of freedom (df = 2), and p-value between audio duration and the corresponding kappa statistics for consistency between CATI first contact with IVR follow up for demographics in Tanzania was r(2) = -0.375, p > 0.10 (two tailed); for audio duration and IVR first contact/CATI follow up was r(2) = -0.449, p > 0.10 (two tailed). The coefficients and associated degrees of freedom (df = 11), and p-value for the correlation between audio duration and kappa statistics for NCD variables were r(11) = -0.052, p > 0.10 (two tailed) for CATI first contact/IVR follow up and r(11) = -0.223, p > 0.10 (two tailed).

## Discussion

This study is one of the few empirical studies that have sought to compare the reliability of survey responses across two different MPS modalities in LMICs. Overall, we observed moderate to high reliability of answers between CATI and IVR for many of the demographic and NCD risk factor variables in Bangladesh and Tanzania, and lower reliability for others. Moreover, the orders of magnitude of kappa statistics were largely similar across the two countries. This study was exploratory in nature and hence we did not set out to prove or disprove any specific hypothesis. Here we offer some reflections on our observations and suggest some possible explanations and ideas, which we hope that other researchers or our team will investigate further.

### Measurement effects

In general, there was higher reliability for responses to demographic questions than to NCD behavioral risk factor questions. This may have two causes: 1) the demographic questions were simpler to respond to due to being shorter questions, and having clearer response options, and 2) questions were easier to understand since the concepts were more familiar to the respondent. We found moderate to high kappa statistics of agreement for current smoking, history of alcohol consumption and having taken six or more drinks in the previous 30 days, having been checked for hypertension or diabetes and having been diagnosed for hypertension. Conversely, kappa statistics for agreement were generally low for history of having been diagnosed with diabetes, low or poor for reports of consumption of weekly fruit and vegetable, and for foods high in salt, as well as for reported vigorous and moderate physical activity in a typical week.

It has been shown that the mode of delivery of a questionnaire can have an important effect on the quality of data [8,20,29,30,32,53]. Of the three main sources of survey error (frame, non-response and measurement), we were mainly concerned with measurement error. Our use of an experimental design, which is rare in mode effects research, was aimed at mitigating some of the potential biases such as selection of participants [54]. In this paper we have shown that MPS participants receiving either an IVR or CATI survey on first contact may provide comparable responses for certain types of questions but not others when re-surveyed using a different survey delivery modality. This suggests that mobile phone survey researchers should be wary of lumping together results collected using different modes, even when collected contemporaneously. Further research may shed light on the extent and characteristics of these mode effects. One option is to compare both MPS delivery modalities to a third reference such as in-person F2F surveys. However, it is worth mentioning that all these survey modalities rely on self-reported behavior and results may not be a true reflection of the respondent’s risk behavior. In other words, there is currently no true ‘gold standard’ against which to compare such MPS self-reported behavioral risk factors. Well designed and well executed F2F surveys asking similar questions, and using additional prompts such as show cards and other ways of validating responses might be considered the next best alternative to compare responses to these behavioral risk factor questions provided they are contemporaneous. There is, however, literature from the United States to suggest that for some sensitive topics, respondents may be more likely to respond honestly when using IVR than CATI or in-person interviews [3,55,56], the phenomenon of social desirability bias [57] as referred to earlier in this paper. Conversely, the possibility of establishing rapport with a respondent that occurs during in-person CATI or F2F interviews often leads to higher completion rates [17]. There is a dearth of research about honesty of survey responses and completion rates for CATI and IVR from LMIC settings, especially sub-Saharan Africa [4,22]. Further research on use of MPS in LMICs will help provide additional clarity on this and other sources of measurement error in these contexts amidst a growing use of mobile phones in social sector surveys.

### Selection effects

Our findings suggest that female respondents, those with lower levels of schooling and rural residents may have higher participation rates for CATI compared to the IVR survey. The confirmation of these observations awaits further research. If confirmed, such findings would be in line with other research that has shown that CATI is more accessible than IVR to a more diverse population [58]. The finding that overall there were fewer female respondents than male across all surveys regardless of first contact or follow up mode in both countries should not come as a surprise given the well-known gender gap in mobile phone ownership and access in LMIC settings [59].

### Explaining similarities and differences

What seems to drive similarity or differences in consistency of responses for demographics and NCD behavioral risk factor questions? We surmise these reasons include: a) length and complexity of the question–proxied in our analysis by the combined number of words in the question and response options; b) nature of response options; c) familiarity of the concept being surveyed depending on the socio-cultural context of the respondent; and d) how clearly the concept being surveyed could be translated into local languages without resorting to complex explanations or use of multiple examples.

Some research has shown that the more words there are in a question and its corresponding response options, the lower the reliability of the response [60]. We might also expect that binary response options of the type, e.g., “press 1 for Yes, press 3 for No” would be easier for respondents to quickly familiarize themselves with during a survey and respond correctly, compared to one with multiple choice.

Questions requiring respondents to type more than one number representing a numeric response such as age or quantity may equally be difficult to comprehend immediately in an IVR naïve population without additional explanation. If a concept is already familiar to the respondent (such as different levels of schooling), although the question may have many words, the cognitive burden may be lower, resulting in higher consistency as assessed by the kappa statistic because respondents may readily identify the response options fitting their own experience. Conversely concepts such as descriptions of physical activity, or fruit and vegetable servings may be harder for respondents to comprehend and simultaneously translate into an answer option on their mobile phone keypad in the limited time available before the system automatically drops the call. Researchers designing mobile phone surveys face a trade-off between having shorter questions, with resulting potential loss of clarity, as well as difficulty in adequately presenting complex information, versus more elaborate explanatory statements preceding questions. The latter option may lead to higher respondent drop-off due to fatigue resulting from a higher cognitive burden.

The IVR repeat function may have been useful for respondents who did not understand a question on hearing it the first time. However, if a respondent accidentally entered an option in the valid range but realized soon after that it was wrong and wanted to change, there was no option to do so. Providing automated confirmatory statements or questions would significantly lengthen the survey and possibly lower completion rates. In addition, in the case of non-response after three repeat questions, the system would hang up. This is different from a F2F or CATI survey where the interviewer can clarify questions and exercise patience until the respondent provides an answer [15,61].

Even though we took the precaution of doing formative assessments first, checked, and double-checked the translations, there are some concepts for which it was hard to find direct local language equivalents, hence resorting to providing numerous examples. This issue arose in both countries due to translation of some of the concepts surveyed from English, in which we designed the original questionnaire, into the local languages. There may not be direct local language equivalents for some of the survey content, or these may not readily translate into the local language. A CATI enumerator may provide some clarifications which is not available in IVR surveys. Unlike F2F surveys such as STEPs which have the possibility to include the use of visual prompts or ‘show-cards’ to express concepts being explored, this is not yet possible when conducting CATI or IVR surveys in LMICs. It is possible that, with the questionnaire formulations used, simply relying on an audio explanation will not have been adequate to convey the correct understanding for some of the more complex concepts. Examples of these with a higher cognitive burden include vigorous or moderate physical activity, fruit or vegetable intake in a typical week and serving sizes, and binge-drinking of alcohol among others. Another concept that does not readily translate into local languages is the concept of urban or rural residence. In LMIC settings, respondents may not readily know if they would consider their area of residence to be urban or rural. To mitigate this we attempted to provide examples of some of the major urban areas in each country. However, for reasons of brevity, it is not feasible to list all the possible urban areas in a country with which a respondent would be familiar enough to make a good judgement of whether her or his own area qualifies to be considered urban or rural. With urban populations at 34% and 31% in Bangladesh and Tanzania, and with relatively high annual urbanization rates in both countries of 2.4 and 2.3 respectively [62], it is possible that a large number of respondents may have been unsure how to respond.

It was interesting to note that, in general, there were higher kappa statistics for comparisons where IVR was the first contact mode with CATI follow up, as opposed to those where CATI first contact was followed by IVR. Although not definitive, this observation warrants further investigation in future studies. One possible explanation could be that people who respond to IVR surveys tend to be better educated so there may have been some ‘creaming’ effect introduced by delivering IVR first. Such respondents probably understood the questions on IVR better the first time and were then more likely to provide the same response when asked again through CATI. The presence and extent of these effects is still open to further investigation.

### Limitations

We recognize that samples of respondents answering CATI or IVR surveys may not have been completely comparable in terms of background population characteristics and associated estimates of specific risk factors, and that different respondents may have answered different questions even when they participated in both surveys. Therefore, non-response error is a possibility because a respondent may have answered a different set of questions in the two surveys. To mitigate this, we only included responses from individuals who participated in both surveys and we only calculated the kappa statistic for each corresponding pair of variables containing data from both surveys. Overall, we feel that the risk of non-response error affecting the findings we have presented is low. Moreover, in our sensitivity analyses to explore the possible effect of different levels of understanding of the survey, when we restricted the analysis of consistency of NCD responses only to those who provided identical answers to demographic variables, this did not substantially change the kappa statistics.

Even though we used an experimental design, underlying differences in survey participants due to differential selection effects may have persisted. These may have included differences in mobile phone ownership or access by age, gender, or geography. This means that our findings may not be generalizable to the broader population of mobile phone users, let alone the wider population. The predominantly male respondents may have partly been due to cultural norms in these settings that may have made female respondents less likely to pick and respond to an unknown caller. One potential solution would have been to publicize the survey widely and providing a masking number. Such a number could help to identify the institution sending the surveys rather than calls coming from an unknown number. This was not possible given the scope of our study and due to potential technical challenges with the delivery platform.

There exists a risk of bias due to priming of respondents by the previous delivery method. We attempted to control for the possibility of priming effects by using a cross-over design, starting with one method e.g., CATI and switching over to IVR and likewise for those who started with IVR switching over after seven days to CATI. Given rarity for some of the variables such as history of being checked or diagnosed with diabetes, these findings should be interpreted with caution: McHugh (2012) recommends that ideally, sample sizes for kappa statistics should not be less than 30 comparisons; in general, we were able to realize or exceed this for first contact for most variables. However, we had anticipated a much lower loss to follow up (20%) than what was observed in practice (ranging from 46% to 70%).

We also recognize there are some key disadvantages of interviewing the same sample twice [63]. First, respondents may be less likely to complete the second contact carefully. Second, the first survey may have made the respondent aware of knowledge gaps that he or she may have filled in the time between the two surveys. Third, the interviewer may rush through the second interview since the respondent may be impatient or the interviewer tired. We feel that the 7-day gap between the two surveys may have helped mitigate some of this. Finally, we had no way of ascertaining the identity of the respondents and cannot be sure that the same respondent answered both surveys. One could add a screening question to check that the person answering the follow up survey is the same one who answered during first contact. We did not include such a question. It is possible that in some cases, such as where phones are shared, the person who responded to the survey the first time may not have been the same one who responded the second time.

## Conclusions

The study found that when respondents are re-interviewed, whether starting with CATI or IVR, the reliability of answers to demographic and NCD variables is similar. The study underscores that researchers need to exercise caution when selecting questions for mobile phone surveys. Our findings show that reliability varies by question. Careful design can help ensure clarity of questions to minimize the cognitive burden for respondents, many of whom may not have prior experience in taking automated surveys. Further research should explore possible differences and determinants of survey reliability between delivery modes and ideally compare both IVR and CATI surveys to in-person face-to-face interviews. In addition, research is needed to better understand factors that influence survey cooperation, completion, refusal and attrition rates across different populations and regions.

## Supporting information

S1 Table Equations used to calculate survey response rates from computer assisted telephone interviews and interactive voice response mobile phone surveys in Bangladesh and Tanzania. (DOCX)

S2 Table Sensitivity analysis for percent agreement and kappa statistics in comparing selected demographics from computer assisted telephone interviews and interactive voice response mobile phone surveys in Bangladesh and Tanzania. (DOCX)

S3 Table Type and length of question and reliability of responses from computer assisted telephone interviews and interactive voice response mobile phone surveys in Bangladesh and Tanzania. (DOCX)

S1 File Bangladesh questionnaire for mobile phone survey on non-communicable disease risk factors in Bangla and English. (PDF)

S2 File Tanzania questionnaire for mobile phone survey on non-communicable disease risk factors in Kiswahili and English. (PDF)

## References

[1] International Telecomunications Union. ICT facts and figures 2017. Itu. 2017; 1–8. 10.1787/9789264202085-5-en

[2] GSMA (b). The Mobile Economy Asia Pacific 2017. GSM Assoc. 2017;6: 1–54.

[3] Ali J, Labrique ABAB, Gionfriddo K, Pariyo G, Gibson DGDG, Pratt B, et al Ethics considerations in global mobile phone-based surveys of noncommunicable diseases: A conceptual exploration. J Med Internet Res. 2017;19: 1–2. 10.2196/jmir.7326 28476723 PMC5438462

[4] Gibson DG, Pereira A, Farrenkopf BA, Labrique AB, Pariyo GW, Hyder AA. Mobile phone surveys for collecting population-level estimates in low-and middle-income countries: A literature review. J Med Internet Res. JMIR Publications Inc.; 2017;19: e139 10.2196/jmir.7428 28476725 PMC5438460

[5] Himelein K, Testaverde M, Turay A, Turay S. The Socio-Economic Impacts of Ebola in Sierra Leone. World Bank Other Oper Stud 2015; Available: http://ideas.repec.org/p/wbk/wboper/22037.html

[6] Figueroa M.E. and JDSBA. Use of SMS-Based Surveys in the Rapid Response to the Ebola Outbreak in Liberia: Opening Community Dialogue. J Heal Commun. 2017; 10.1080/10810730.2016.1224279 28854132

[7] Larmarange J, Kassoum O, Kakou É, Fradier Y, Sika L, Danel C. Faisabilité et représentativité d’une enquête téléphonique avec échantillonnage aléatoire de lignes mobiles en Côte d’Ivoire. Population (Paris). 2016;71: 121 10.3917/popu.1601.0121

[8] Leo B, Morello R, Mellon J, Peixoto T, Davenport S. Do Mobile Phone Surveys Work in Poor Countries? CGD Work Pap. 2015; 1–65. 10.2139/ssrn.2597885

[9] Dabalen A, Etang A, Hoogeveen J, Mushi E, Schipper Y, von Engelhardt J. Mobile Phone Panel Surveys in Developing Countries: a practical guide for microdata collection [Internet]. The World Bank; 2016 10.1596/978-1-4648-0904-0

[10] Diedhiou A, Gilroy KE, Cox CM, Duncan L, Koumtingue D, Pacque-Margolis S, et al Successful mLearning Pilot in Senegal: Delivering Family Planning Refresher Training Using Interactive Voice Response and SMS. Glob Heal Sci Pract. 2015;3: 305–321. 10.9745/GHSP-D-14-00220 26085026 PMC4476867

[11] Long L, Pariyo G, Kallander K. Digital Technologies for Health Workforce Development in. Glob Heal Sci Pract. 2018; 1–8. 10.9745/GHSP-D-18-00167 PMC6203417 30305338

[12] Pariyo GW, Wosu AC, Gibson DG, Labrique AB, Ali J, Hyde AA. Moving the agenda on noncommunicable diseases: Policy implications of mobile phone surveys in low and middle-income countries. J Med Internet Res. 2017;19 10.2196/jmir.7302 28476720 PMC5438456

[13] Institute for Health Metrics and Evaluation. Global Burden of Disease Study 2015. In: Seattle, United States: Institute for Health Metrics and Evaluation (IHME), 2016. For terms and conditions of use, please visit http://www.healthdata.org/about/terms-and-conditions. [Internet]. 2016. Available: http://vizhub.healthdata.org/gbd-compare/

[14] United Nations. Sustainable Development Goals—Health. In: Sustainable Development Goals [Internet]. 2018 [cited 12 Oct 2018]. Available: https://www.un.org/sustainabledevelopment/health/

[15] Kelly J. Computer-Assisted Telephone Interviewing (CATI). In: Encyclopedia of Survey Research Methods In: Lavrakas PJ, editor. Encyclopedia of Survey Research Methods. AM. Vol. 1 2455 Teller Road, Thousand Oaks California 91320 United States of America: Sage Publications, Inc.; 2008 pp. 122–125. 10.4135/9781412963947

[16] Leo B, Morello R. Asking What the People Want: Using Mobile Phone Surveys to Identify Citizen Priorities Working Paper 418 August 2015. 2015;

[17] Corkrey R, Parkinson L. A comparison of four computer-based telephone interviewing methods: Getting answers to sensitive questions [Internet]. Behavior Research Methods, Instruments, and Computers. Springer-Verlag; 2002 pp. 354–363. 10.3758/BF03195463 12395551

[18] Currivan DB. Interactive Voice Response (IVR). In: Encyclopedia of Survey Research Methods In: Lavrakas PJ, editor. Encyclopedia of Survey Research Methods. Thousand Oaks: Sage Publications, Inc; 2008 pp. 343–344. 10.4135/9781412963947

[19] Greenleaf A., Vogel L. Interactive Voice Response Technology for Data Collection in Sub-Saharan Africa. Br Viamo. 2018;

[20] Christian LM, Foster KN. Multi-Mode Surveys. In: Encyclopedia of Survey Research Methods In: Lavrakas PJ, editor. Encyclopedia of Survey Research Methods. Thousand Oaks: Sage Publications, Inc.; 2008 pp. 487–488. 10.4135/9781412963947

[21] Biemer PP. Total survey error: Design, implementation, and evaluation. Public Opin Q. 2010;74: 817–848. 10.1093/poq/nfq058

[22] Greenleaf AR, Gibson DG, Khattar C, Labrique AB, Pariyo GW. Building the evidence base for remote data collection in low and middle-income countries: Comparing reliability and accuracy across survey modalities. J Med Internet Res Internet Res. JMIR Publications Inc.; 2017;19: e140 10.2196/jmir.7331 28476728 PMC5438451

[23] Labrique A, Blynn E, Ahmed S, Gibson D, Pariyo G, Hyder AA. Health surveys using mobile phones in developing countries: Automated active strata monitoring and other statistical considerations for improving precision and reducing biases. J Med Internet Res. Journal of Medical Internet Research; 2017;19: e121 10.2196/jmir.7329 28476726 PMC5438457

[24] Tourangeau R, Rips LJ, Rasinski KA. The psychology of survey response. Cambridge University Press; 2000.

[25] Tourangeau R, Yan T. Sensitive Questions in Surveys. Psychol Bull. 2007;133: 859–883. 10.1037/0033-2909.133.5.859 17723033

[26] Kreuter F, Presser S, Tourangeau R. Social desirability bias in CATI, IVR, and web surveys: The effects of mode and question sensitivity. Public Opin Q. 2008;72: 847–865. 10.1093/poq/nfn063

[27] Andrew Mercer; Kyley McGeeney; Nancy Mathiowetz; Ruth Igielnik; Scott Keeter. From Telephone to the Web: The Challenge of Mode of Interview Effects In Public Opinion Polls [Internet]. Methods. 2015 Available: http://www.pewresearch.org/methods/2015/05/13/from-telephone-to-the-web-the-challenge-of-mode-of-interview-effects-in-public-opinion-polls/

[28] Simon HA. A study of Decision-Making Processes in Administrative Organiztions Administrative Behavior. Third Edit The Free Press, Collier Macmillan Publishers 1976; 1997. p. 31. Available: https://www.google.com/search?q=Simon+H+A+(1945)%3B+Administrative+Behavior.+A+Study+o+f+Decision-Making+Processes+in+Administrative+Organization.+Third+Edition.+The+Free+Press,+Collier+Macmillan+Publishers.+1976.&source=univ&tbm=shop&tbo=u&sa=X&ved=0ahUK

[29] Ongena YP, Dijkstra W. A Model of Cognitive Processes and Conversational Principles in Survey Interview Interaction. Appl Cogn Psychol. 2007;21: 145–163. 10.1002/acp.1334

[30] Groves RM. Theories and Methods of Telephone Surveys. Annu Rev Sociol. 1990;16: 221–240. 10.1146/annurev.so.16.080190.001253

[31] Green M, Krosnick J, Holbrook A. The survey response process in telephone and face-to-face surveys: Differences in respondent satisficing and social desirability response bias [Internet]. …://Www. Psy. Ohiostate. Edu/Social …. 2001 Available: https://pdfs.semanticscholar.org/d4d8/42f48a2109e094fc6590f69e386940e2e32e.pdf

[32] Krosnick JA, Narayan S, Smith WR. Satisficing in surveys: Initial evidence New Dir Eval. John Wiley & Sons, Ltd; 1996;1996: 29–44. 10.1002/ev.1033

[33] Haeger H, Lambert AD. Using Cognitive Interviews to Improve Survey Instruments. Annu forum Assoc Institutional Res. 2012; 15 Available: http://cpr.indiana.edu/uploads/AIR2012 Cognitive Interviews.pdf

[34] Robling MR, Ingledew DK, Greene G, Sayers A, Shaw C, Sander L, et al Applying an extended theoretical framework for data collection mode to health services research [Internet]. BMC Health Services Research 2010 10.1186/1472-6963-10-180 PMC2903587 20576131

[35] Brick JM. Random-Digit Dialing (RDD). In: Encyclopedia of Survey Research Methods In: Lavrakas PJ, editor. Encyclopedia of Survey Research Methods. Thousand Oaks: Sage Publications, Inc; 2008 pp. 676–678. 10.4135/9781412963947

[36] Parkin M. Priming. In: Encyclopedia of Survey Research Methods In: Lavrakas PJ, editor. Encyclopedia of Survey Research Methods. Thousand Oaks: Sage Publications, Inc.; 2008 10.4135/9781412963947

[37] Hjortskov M. Priming and context effects in citizen satisfaction surveys. Public Adm. 2017;95: 912–926. 10.1111/padm.12346

[38] Garbarski D, Schaeffer NC, Dykema J. The effects of response option order and question order on self-rated health. Qual Life Res. NIH Public Access; 2015;24: 1443–53. 10.1007/s11136-014-0861-y 25409654 PMC4440847

[39] Gibson DG, Pariyo GW, Wosu AC, Greenleaf AR, Ali J, Ahmed S, et al Evaluation of Mechanisms to Improve Performance of Mobile Phone Surveys in Low- and Middle-Income Countries: Research Protocol. JMIR Res Protoc. 2017;6: e81 10.2196/resprot.7534 28476729 PMC5438454

[40] World Health Organization. The WHO STEPwise approach to Surveillance of noncommunicable diseases (STEPS). 2003; 43. Available: http://www.who.int/ncd_surveillance/en/steps_framework_dec03.pdf

[41] Riley L, Guthold R, Cowan M, Savin S, Bhatti L, Armstrong T, et al The world health organization STEPwise approach to noncommunicable disease risk-factor surveillance: Methods, challenges, and opportunities. Am J Public Health. 2016;106: 74–78. 10.2105/AJPH.2015.302962 26696288 PMC4695948

[42] Global Adult Tobacco Survey Collaborative Group. Tobacco Questions for Surveys: A Subset of Key Questions from the Global Adult Tobacco Survey (GATS) [Internet]. 2nd Editio Atlanta, GA: Centers for Disease Control and Prevention; 2011 Available: http://www.who.int/tobacco/surveillance/en_tfi_tqs.pdf?ua=1

[43] Centers for Disease Control. Behavioral Risk Factor Surveillance System [Internet]. About BRFSS. 2015 Dec. Available: http://www.cdc.gov/brfss/

[44] Johnson A, Kelly F, Stevens S. Modular survey design for mobile devices. 2012 CASRO Online Conference. 2012. Available: https://c.ymcdn.com/sites/www.casro.org/resource/collection/E270CC91-6B72-4C37-BCC0-5503CBB66C55/Paper_-_Frank_Kelly_and_Alex_Johnson_-_Lightspeed_Research_and_Kantar_Operations.pdf

[45] West BT, Axinn WG. Evaluating a Modular Design Approach to Collecting Survey Data Using Text Messages HHS Public Access. Surv Res Methods. NIH Public Access; 2015;9: 111–123. 10.18148/srm/2015.v9i2.6135 26322137 PMC4551499

[46] Open Knowledge Foundation. CSV–Comma Separated Values. In: Sustainability of Digital Formats [Internet]. 2015 [cited 8 Nov 2018]. Available: https://www.loc.gov/preservation/digital/formats/fdd/fdd000323.shtml

[47] StataCorp. Stata Statistical Software: Release 14 [Internet]. College Station, TX: StataCorp LP 2015 Available: https://www.stata.com/stata14/

[48] Cohen J. A Coefficient of Agreement for Nominal Scales Educ Psychol Meas. Sage PublicationsSage CA: Thousand Oaks, CA; 1960;20: 37–46. 10.1177/001316446002000104

[49] Mabmud SM. Cohen’s Kappa. In: Encyclopedia of Research Design In: Salkind NJ, editor. Encyclopedia of Research Design. Thousand Oaks: SAGE Publications, Inc.; 2010 10.4135/9781412961288

[50] McHugh ML. Interrater reliability: the kappa statistic. Biochem medica. 2012;22: 276–82. Available: http://www.ncbi.nlm.nih.gov/pubmed/23092060 PMC3900052 23092060

[51] Landis JR, Koch GG. The Measurement of Observer Agreement for Categorical Data. Biometrics. 1977;33: 159 10.2307/2529310 843571

[52] The American Association for Public Opinion Research. Standard Definitions: Final Dispositions of Case Codes and Outcome Rates for Surveys [Internet]. 9th edition AAPOR 2016 Available: https://www.aapor.org/AAPOR_Main/media/publications/Standard-Definitions20169theditionfinal.pdf

[53] Tourangeau R, Rips LJ, Rasinski K. The Psychology of Survey Response [Internet]. Cambridge: Cambridge University Press; 2000 10.1017/CBO9780511819322

[54] Bowling A. Mode of questionnaire administration can have serious effects on data quality. J Public Health (Bangkok). 2005;27: 281–291. 10.1093/pubmed/fdi031 15870099

[55] Midanik LT, Greenfield TK. Interactive Voice Response Versus Computer-Assisted Telephone Interviewing (CATI) Surveys and Sensitive Questions: The 2005 National Alcohol Survey. J Stud Alcohol Drugs. 2008;69: 580–588. 10.15288/jsad.2008.69.580 18612574

[56] Ali J, DiStefano MJ, Coates McCall I, Gibson DG, Al Kibria GM, Pariyo GW, et al Ethics of mobile phone surveys to monitor non-communicable disease risk factors in low- and middle-income countries: A global stakeholder survey Glob Public Health. Taylor & Francis; 2019; 1–15. 10.1080/17441692.2019.1566482 30628548

[57] Spector PE. Social Desirability Bias. In: The SAGE Encyclopedia of Social Science Research Methods In: Michael S. Lewis-Beck AB& TFL, editor. The SAGE Encyclopedia of Social Science Research Methods. Thousand Oaks: Sage Publications, Inc.; 2004 p. 1045 10.4135/9781412950589

[58] Ballivian Amparo, João Pedro Azevedo Will Durbin. Using Mobile Phones for High-Frequency Data Collection. Toninelli D, Pinter R, Pedraza P (Eds), Mob Res methods Oppor challenges Mob Res Methodol. 2015; 21–39. 10.5334/bar.c

[59] GSMA. Bridging the gender gap: Mobile access and usage in low-and middle-income countries [Internet]. GSMA; 2015 Available: www.altaiconsulting.com

[60] Alwin DF, Beattie BA. The kiss principle in survey design: Question length and data quality Sociol Methodol. SAGE PublicationsSage CA: Los Angeles, CA; 2016;46: 121–152. 10.1177/0081175016641714

[61] Olsen R, Sheets C. Voice over Internet Protocol (VoIP) and the Virtual Computer-Assisted Telephone Interview (CATI) Facility. In: Encyclopedia of Survey Research Methods In: Lavrakas PJ, editor. Encyclopedia of Survey Research Methods. Thousand Oaks: Sage Publications, Inc.; 2018 pp. 951–952. 10.4135/9781412963947

[62] UN. World Urbanization Prospects [Internet]. Sustainable Development. 2015. Available: https://esa.un.org/unpd/wup/publications/files/wup2014-highlights.pdf

[63] Schreiner M. There’s No Place Like Home (How the Interview Method Affects Results with the Progress out of Poverty Index). 2015.

