# Supplementary material for: Does mobile phone survey method matter? Reliability of computer-assisted telephone interviews and interactive voice response non-communicable diseases risk factor surveys in low and middle income countries
Source: PLoS One. 2019 Apr 10;14(4):e0214450. doi: 10.1371/journal.pone.0214450 (PMC6457489; doi:10.1371/journal.pone.0214450)
Supplement: S1 Table — (DOCX) [file pone.0214450.s001.docx]

**S1 Table. Equations used to calculate survey response rates from computer assisted telephone interviews and interactive voice response mobile phone surveys in Bangladesh and Tanzania.**

| **AAPOR* Category** | **Equation** |
| --- | --- |
| Contact Rate # 1 | ($\left( I+P \right)+R+O)/$($\left( I+P \right)+R+O+NC+ (UH+UO))$ |
| Response Rate #2 | ($I+P)/$($\left( I+P \right)+\left( R+O+ NC \right)+ (UH+UO))$ |
| Refusal rate #1 | *R*/($\left( I+P \right)+\left( R+O+NC \right)+ (UH+UO))$ |
| Cooperation rate #2 | (I + P)/($I+P+R+O)$ |

*AAPOR = American Association for Public Opinion Research.

Abbreviations: I – Interview; P – Partial Interview; R – Refusal/Break-off; O – Other; NC – Non-contact; UH – Unknown Household/Number; UO – Unknown Other;

Reference: The American Association for Public Opinion Research. Standard Definitions: Final Dispositions of Case Codes and Outcome Rates for Surveys [Internet]. 9th edition. AAPOR. 2016 [cited 2019 Feb 28]. Available from: <https://www.aapor.org/AAPOR_Main/media/publications/Standard-Definitions20169theditionfinal.pdf>
