# Supplementary material for: Does mobile phone survey method matter? Reliability of computer-assisted telephone interviews and interactive voice response non-communicable diseases risk factor surveys in low and middle income countries
Source: PLoS One. 2019 Apr 10;14(4):e0214450. doi: 10.1371/journal.pone.0214450 (PMC6457489; doi:10.1371/journal.pone.0214450)
Supplement: S2 Table — (DOCX) [file pone.0214450.s002.docx]

Supporting tables

S2 Table A. Sensitivity analysis for percent agreement and kappa Statistics in comparing selected demographics from computer assisted telephone interviews and interactive voice response mobile phone surveys in Bangladesh and Tanzania.

|  | **Bangladesh** | | | | | | **Tanzania** | | | | | |
| --- | --- | --- | --- | --- | --- | --- | --- | --- | --- | --- | --- | --- |
|  | **Dropped incomplete data** | | **Only complete demographics + at least 1 NCD module** | | **Same response on demographics in both surveys (age, gender, schooling)** | | **Dropped incomplete data** | | **Only complete demographics + at least 1 NCD module** | | **Same response on demographics in both surveys (age, gender, schooling)** | |
|  | **Arm 1** | **Arm 2** | **Arm 1** | **Arm 2** | **Arm 1** | **Arm 2** | **Arm 1** | **Arm 2** | **Arm 1** | **Arm 2** | **Arm 1** | **Arm 2** |
|  | **IVR Follow up (**CATI🡪IVR**)** | **CATI Follow up (**IVR🡪CATI**)** | **IVR Follow up (**CATI🡪IVR**)** | **CATI Follow up (**IVR🡪CATI**)** | **IVR Follow up (**CATI🡪IVR**)** | **CATI Follow up (**IVR🡪CATI**)** | **IVR Follow up (**CATI🡪IVR**)** | **CATI Follow up (**IVR🡪CATI**)** | **IVR Follow up (**CATI🡪IVR**)** | **CATI Follow up (**IVR🡪CATI**)** | **IVR Follow up (**CATI🡪IVR**)** | **CATI Follow up (**IVR🡪CATI**)** |
| 1. **Age** | (n=195) | (n=342) | (n=162) | (n=340) | (n=60) | (n=155) | (n=161) | (n=335) | (n=164) | (n=332) | (n=73) | (n=20) |
| **Expected Agreement** | 4.11% | 5.87% | 4.53% | 5.82% | 6.44% | 7.04% | 3.80% | 4.26% | 3.96% | 4.28% | 4.82% | 10.0% |
| **Observed Agreement** | 46.15% | 63.16% | 54.12% | 62.94% | 100.0% | 100.0% | 56.67% | 69.55% | 57.32% | 70.18% | 100.0% | 100.0% |
| **Kappa statistic** | 0.4385 | 0.6086 | 0.5194 | 0.6065 | 1.0 | 1.0 | 0.5496 | **0.6820** | 0.556 | **0.6885** | 1.0 | 1.0 |
| **S.E.** | 0.0136 | 0.0132 | 0.0163 | 0.0132 | 0.0331 | 0.0215 | 0.0146 | 0.0114 | 0.0156 | 0.0115 | 0.0261 | 0.0726 |
| 1. **Gender** | (n=180) | (n=342) | (n=170) | (n=340) | (n=60) | (n=155) | (n=173) | (n=335) | (n=164) | (n=332) | (n=73) | (n=20) |
| **Expected Agreement** | 57.35% | 82.29% | 57.77% | 82.20% | 54.50% | 77.52% | 60.69% | 60.98% | 62.80% | 60.76% | 64.27% | 58.0% |
| **Observed Agreement** | 92.78% | 97.95% | 93.53% | 97.94% | 100.0% | 100.0% | 95.95% | 95.22% | 95.73% | 95.18% | 100.0% | 100.0% |
| **Kappa statistic** | **0.8307** | **0.8844** | **0.8468** | **0.8843** | 1.0 | 1.0 | **0.8971** | **0.8776** | **0.8852** | **0.8772** | 1.0 | 1.0 |
| **S.E.** | 0.0736 | 0.0533 | 0.0757 | 0.0534 | 0.1291 | 0.0803 | 0.0760 | 0.0546 | 0.0781 | 0.0549 | 0.1170 | 0.2236 |
| 1. **Urban/rural residence** | (n=178) | (n=342) | (n=170) | (n=340) | (n=60) | (n=155) | (n=171) | (n=335) | (n=164) | (n=332) | (n=73) | (n=20) |
| **Expected Agreement** | 51.40% | 51.74% | 51.04% | 51.76% | 49.94% | 51.77% | 53.10% | 52.46% | 52.91% | 52.40% | 55.17% | 50.0% |
| **Observed Agreement** | 86.52% | 87.72% | 87.65% | 87.65% | 86.67% | 89.03% | 77.78% | 79.70% | 78.05% | 80.12% | 76.41% | 60.0% |
| **Kappa statistic** | **0.7226** | **0.7455** | **0.7477** | **0.7439** | **0.7336** | **0.7726** | 0.5262 | 0.5730 | 0.5339 | 0.5824 | 0.4805 | 0.2 |
| **S.E.** | 0.0749 | 0.0541 | 0.0766 | 0.0542 | 0.1288 | 0.0800 | 0.0763 | 0.0542 | 0.0779 | 0.0543 | 0.1157 | 0.2049 |
| 1. **Schooling** | (n=179) | (n=340) | (n=170) | (n=340) | (n=60) | (n=155) | (n=168) | (n=195) | (n=164) | (n=192) | (n=73) | (n=20) |
| **Expected Agreement** | 21.85% | 22.41% | 21.95% | 22.41% | 55.34% | 25.17% | 38.08% | 15.57% | 37.86% | 15.78% | 37.29% | 55.5% |
| **Observed Agreement** | 62.01% | 69.12% | 61.76% | 69.12% | 83.64% | 100.0% | 79.76% | 17.95% | 79.27% | 18.23% | 100.0% | 100.0% |
| **Kappa statistic** | 0.5139 | 0.6020 | 0.5101 | 0.6020 | 0.6336 | 1.0 | **0.6732** | 0.0282 | **0.664** | 0.0291 | 1.0 | 1.0 |
| **S.E.** | 0.0385 | 0.0284 | 0.0396 | 0.0284 | 0.1347 | 0.0449 | 0.0554 | 0.0147 | 0.0558 | 0.0151 | 0.0858 | 0.1917 |

CATI – Computer Assisted Telephone Interviews; IVR – Interactive Voice Response; CATI🡪IVR indicates IVR as follow up mode (after CATI first contact). IVR🡪CATI indicates CATI as follow up mode (after IVR first contact).

NB: The kappa-statistic measure of agreement is scaled to be 0 when the amount of agreement is what would be expected to be observed by chance; -1 would represent perfect disagreement; and +1 would represent perfect agreement. For intermediate values, Landis and Koch (1977a, 165) suggest the following interpretations: below 0.0 Poor; 0.00 – 0.20 Slight; 0.21 – 0.40 Fair; 0.41 – 0.60 Moderate; **0.61 – 0.80 Substantial, 0.81 – 1.00 Almost perfect. Bolded kappa statistics in the table represent the ‘substantial’, and ‘almost perfect’ agreement categories.**

S2 Table B. Sensitivity analysis for percent agreement and kappa Statistics in comparing selected non-communicable disease risk factors from computer assisted telephone interviews and interactive voice response mobile phone surveys in Bangladesh and Tanzania.

|  | **Bangladesh** | | | | | | **Tanzania** | | | | | |
| --- | --- | --- | --- | --- | --- | --- | --- | --- | --- | --- | --- | --- |
|  | **Dropped incomplete data** | | **Only complete demographics + at least 1 NCD module** | | **Same response on demographics in both surveys (age, gender, schooling)** | | **Dropped incomplete data** | | **Only complete demographics + at least 1 NCD module** | | **Same response on demographics in both surveys (age, gender, schooling)** | |
|  | **Arm 1** | **Arm 2** | **Arm 1** | **Arm 2** | **Arm 1** | **Arm 2** | **Arm 1** | **Arm 2** | **Arm 1** | **Arm 2** | **Arm 1** | **Arm 2** |
|  | **IVR Follow up (**CATI🡪IVR**)** | **CATI Follow up (**IVR🡪CATI**)** | **IVR Follow up (**CATI🡪IVR**)** | **CATI Follow up (**IVR🡪CATI**)** | **IVR Follow up (**CATI🡪IVR**)** | **CATI Follow up (**IVR🡪CATI**)** | **IVR Follow up (**CATI🡪IVR**)** | **CATI Follow up (**IVR🡪CATI**)** | **IVR Follow up (**CATI🡪IVR**)** | **CATI Follow up (**IVR🡪CATI**)** | **IVR Follow up (**CATI🡪IVR**)** | **CATI Follow up (**IVR🡪CATI**)** |
| 1. **Smoking tobacco Currently** | (n=154) | (n=326) | (n=150) | (n=326) | (n=53) | (n=150) | (n=149) | (n=318) | (n=146) | (n=318) | (n=68) | (n=19) |
| **Expected Agreement** | 46.42% | 42.54% | 45.97% | 42.54% | 60.06% | 47.85% | 82.33% | 82.92% | 81.99% | 82.92% | 90.03% | 85.04% |
| **Observed Agreement** | 83.12% | 82.21% | 83.33% | 82.21% | 88.68% | 90.0% | 89.26% | 91.51% | 89.04% | 91.51% | 92.65% | 94.74% |
| **Kappa statistic** | **0.6849** | **0.6904** | **0.6915** | **0.6904** | **0.7166** | **0.8082** | 0.3925 | 0.5030 | 0.3917 | 0.5030 | 0.2625 | **0.6481** |
| **S.E.** | 0.0610 | 0.0416 | 0.0620 | 0.0416 | 0.1052 | 0.0625 | 0.0585 | 0.0416 | 0.0591 | 0.0416 | 0.0853 | 0.1672 |
| 1. **Positive history of alcohol consumption last 30 days** | (n=18) | (n=50) | (n=18) | (n=50) | (n=6) | (n=20) | (n=41) | (n=84) | (n=40) | (n=84) | (n=20) | (n=5) |
| **Expected Agreement** | 84.57% | 65.36% | 84.57% | 65.36% | -- | 65.00% | 50.21% | 49.74% | 50.00% | 49.74% | 50.50% | 52.00% |
| **Observed Agreement** | 94.44% | 84.00% | 94.44% | 84.00% | -- | 95.00% | 82.93% | 88.10% | 82.50% | 88.10% | 90.00% | 100.00% |
| **Kappa statistic** | **0.6400** | 0.5381 | **0.6400** | 0.5381 | -- | **0.8571** | **0.6571** | **0.7631** | **0.6500** | **0.7631** | **0.7980** | **1.0000** |
| **S.E.** | 0.2199 | 0.1376 | 0.2199 | 0.1376 | -- | 0.2213 | 0.1545 | 0.1060 | 0.1563 | 0.1060 | 0.2236 | 0.4472 |
| 1. **Positive history of alcohol - 6 or more drinks last 30 days** | (n=0) | (n=7) | (n=0) | (n=7) | (n=0) | (n=4) | (n=19) | (n=33) | (n=18) | (n=33) | (n=10) | (n=3) |
| **Expected Agreement** | - | 51.02% | - | 51.02% | -- | 50.00% | 56.23% | 47.93% | 54.94% | 47.93% | 66.00% | 44.44% |
| **Observed Agreement** | - | 42.86% | - | 42.86% | -- | 25.00% | 78.95% | 78.79% | 77.78% | 78.79% | 80.00% | 66.67% |
| **Kappa statistic** | - | -0.1667 | - | -0.1667 | -- | -0.5000 | 0.5190 | 0.5926 | 0.5068 | 0.5926 | 0.4118 | 0.4000 |
| **S.E.** | - | 0.3780 | - | 0.3780 | -- | 0.4330 | 0.2227 | 0.1590 | 0.2284 | 0.1590 | 0.2557 | 0.4619 |
| 1. **Any fruit in typical week** | (n=162) | (n=339) | (n=158) | (n=339) | (n=57) | (n=154) | (n=162) | (n=330) | (n=159) | (n=330) | (n=72) | (n=20) |
| **Expected Agreement** | 87.31% | 81.38% | 87.01% | 81.38% | 88.46% | 85.71% | 81.70% | 82.62% | 81.40% | 82.62% | 87.04% | 81.50% |
| **Observed Agreement** | 88.89% | 86.14% | 88.61% | 86.14% | 91.23% | 90.26% | 88.27% | 89.09% | 88.05% | 89.09% | 91.67% | 90.00% |
| **Kappa statistic** | 0.1243 | 0.2552 | 0.1228 | 0.2552 | 0.2400 | 0.3186 | 0.3590 | 0.3724 | 0.3576 | 0.3724 | 0.3571 | 0.4595 |
| **S.E.** | 0.0771 | 0.0398 | 0.0780 | 0.0398 | 0.1309 | 0.0590 | 0.0785 | 0.0480 | 0.0793 | 0.0480 | 0.1151 | 0.1881 |
| 1. **Any vegetable in typical week** | (n=154) | (n=324) | (n=151) | (n=324) | (n=55) | (n=151) | (n=161) | (n=331) | (n=158) | (n=331) | (n=72) | (n=20) |
| **Expected Agreement** | 97.43% | 89.99% | 98.68% | 89.99% | 96.43% | 90.29% | 89.90% | 88.00% | 90.31% | 88.00% | 90.74% | 86.00% |
| **Observed Agreement** | 98.70% | 91.67% | 97.38% | 91.67% | 100.0% | 92.72% | 90.68% | 92.75% | 91.14% | 92.75% | 93.06% | 95.00% |
| **Kappa statistic** | 0.4951 | 0.1678 | 0.4950 | 0.1678 | 1.0 | 0.2494 | 0.0772 | 0.3958 | 0.0852 | 0.3958 | 0.2500 | **0.6429** |
| **S.E.** | 0.0696 | 0.0308 | 0.0702 | 0.0308 | 0.1348 | 0.0538 | 0.0711 | 0.0503 | 0.0732 | 0.0503 | 0.1165 | 0.2089 |
| 1. **Consumption of processed foods high in salt (always/sometimes)** | (n=154) | (n=338) | (n=151) | (n=338) | (n=54) | (n=154) | (n=151) | (n=329) | (n=148) | (n=329) | (n=71) | (n=20) |
| **Expected Agreement** | 13.64% | 11.77% | 13.78% | 11.77% | 19.10% | 13.48% | 32.13% | 32.86% | 32.19% | 32.86% | 37.85% | 30.25% |
| **Observed Agreement** | 12.99% | 12.72% | 13.25% | 12.72% | 16.67% | 12.99% | 40.40% | 41.95% | 39.86% | 41.95% | 47.89% | 30.00% |
| **Kappa statistic** | -0.0076 | 0.0108 | -0.0062 | 0.0108 | -0.0301 | -0.0058 | 0.1218 | 0.1353 | 0.1132 | 0.1353 | 0.1615 | -0.0036 |
| **S.E.** | 0.0216 | 0.0123 | 0.0221 | 0.0123 | 0.0437 | 0.0189 | 0.0477 | 0.0348 | 0.0479 | 0.0348 | 0.0798 | 0.1303 |
| 1. **Effort to limit salt in diet** | (n=154) | (n=337) | (n=151) | (n=337) | (n=54) | (n=153) | (n=150) | (n=328) | (n=147) | (n=328) | (n=70) | (n=20) |
| **Expected Agreement** | 39.02% | 47.57% | 39.26% | 47.57% | 37.65% | 47.79% | 47.75% | 48.13% | 47.93% | 48.13% | 49.10% | 40.00% |
| **Observed Agreement** | 43.51% | 60.53% | 43.71% | 60.53% | 46.30% | 63.40% | 52.00% | 59.45% | 51.70% | 59.45% | 58.57% | 45.00% |
| **Kappa statistic** | 0.0736 | 0.2473 | 0.0732 | 0.2473 | 0.1386 | 0.2990 | 0.0813 | 0.2182 | 0.0723 | 0.2182 | 0.1860 | 0.0833 |
| **S.E.** | 0.0507 | 0.0490 | 0.0515 | 0.0490 | 0.0813 | 0.0732 | 0.0736 | 0.0512 | 0.0744 | 0.0512 | 0.1113 | 0.1479 |
| 1. **Ever checked high blood pressure** | (n=158) | (n=332) | (n=155) | (n=332) | (n=55) | (n=154) | (n=151) | (n=328) | (n=149) | (n=328) | (n=69) | (n=20) |
| **Expected Agreement** | 50.77% | 49.66% | 50.89% | 49.66% | 53.09% | 45.59% | 51.45% | 56.92% | 51.49% | 56.92% | 49.93% | 62.50% |
| **Observed Agreement** | 83.54% | 76.81% | 83.23% | 76.81% | 83.64% | 81.82% | 75.50% | 85.37% | 75.17% | 85.37% | 79.71% | 90.00% |
| **Kappa statistic** | **0.6657** | 0.5393 | **0.6584** | 0.5393 | **0.6512** | **0.6393** | 0.4953 | **0.6603** | 0.4881 | **0.6603** | 0.5948 | **0.7333** |
| **S.E.** | 0.0789 | 0.0540 | 0.0796 | 0.0540 | 0.1339 | 0.0793 | 0.0805 | 0.0552 | 0.0810 | 0.0552 | 0.1196 | 0.2236 |
| 1. **History of diagnosis of high blood pressure/taking HT medications** | (n=76) | (n=123) | (n=75) | (n=123) | (n=30) | (n=63) | (n=43) | (n=79) | (n=42) | (n=79) | (n=26) | (n=4) |
| **Expected Agreement** | 69.74% | 68.59% | 70.45% | 68.59% | 70.22% | 65.43% | 75.99% | 64.69% | 90.48% | 64.69% | 73.67% | 62.50% |
| **Observed Agreement** | 82.89% | 91.87% | 84.00% | 91.87% | 80.00% | 93.65% | 90.70% | 87.34% | 75.51% | 87.34% | 92.31% | 100.00% |
| **Kappa statistic** | 0.4348 | **0.7412** | 0.4585 | **0.7412** | 0.3284 | **0.8163** | **0.6126** | **0.6416** | **0.6111** | **0.6416** | **0.7079** | **1.0000** |
| **S.E.** | 0.1007 | 0.0902 | 0.1030 | 0.0902 | 0.1353 | 0.1260 | 0.1525 | 0.1113 | 0.1543 | 0.1113 | 0.1876 | 0.5000 |
| 1. **Ever checked diabetes** | (n=161) | (n=335) | (n=157) | (n=335) | (n=55) | (n=154) | (n=153) | (n=329) | (n=150) | (n=329) | (n=69) | (n=20) |
| **Expected Agreement** | 58.92% | 62.36% | 58.31% | 62.36% | 55.34% | 58.74% | 59.86% | 62.56% | 60.08% | 62.56% | 55.95% | 67.50% |
| **Observed Agreement** | 85.71% | 82.69% | 85.35% | 82.69% | 83.64% | 85.06% | 79.08% | 87.84% | 80.67% | 87.84% | 81.16% | 80.00% |
| **Kappa statistic** | **0.6523** | 0.5400 | **0.6486** | 0.5400 | **0.6336** | **0.6381** | 0.4789 | **0.6753** | 0.5157 | **0.6753** | 0.5722 | 0.3846 |
| **S.E.** | 0.0788 | 0.0544 | 0.0798 | 0.0544 | 0.1347 | 0.0798 | 0.0793 | 0.0551 | 0.0790 | 0.0551 | 0.1198 | 0.2128 |
| 1. **History of diagnosis of diabetes/taking diabetes medications** | (n=35) | (n=55) | (n=35) | (n=55) | (n=14) | (n=33) | (n=26) | (n=62) | (n=26) | (n=62) | (n=16) | (n=2) |
| **Expected Agreement** | 59.02% | 65.62% | 59.02% | 65.62% | 66.33% | 66.39% | 92.31% | 83.71% | 92.31% | 83.71% | 93.75% | -- |
| **Observed Agreement** | 88.57% | 92.73% | 88.57% | 92.73% | 85.71% | 93.94% | 92.31% | 88.71% | 92.31% | 88.71% | 93.75% | -- |
| **Kappa statistic** | **0.7211** | **0.7885** | **0.7211** | **0.7885** | 0.5758 | **0.8197** | 0.0000 | 0.3067 | 0.00 | 0.3067 | 0.0000 | -- |
| **S.E.** | 0.1674 | 0.1318 | 0.1674 | 0.1318 | 0.2673 | 0.1712 | 0.0000 | 0.1213 | 0.00 | 0.1213 | 0.0000 | -- |
| 1. **Any vigorous physical activity in typical week** | (n=167) | (n=338) | (n=159) | (n=338) | (n=52) | (n=155) | (n=153) | (n=329) | (n=151) | (n=329) | (n=70) | (n=20) |
| **Expected Agreement** | 46.69% | 48.42% | 47.16% | 48.42% | 46.30% | 48.22% | 58.17% | 60.77% | 58.78% | 60.77% | 60.78% | 59.00% |
| **Observed Agreement** | 62.28% | 65.68% | 63.52% | 65.68% | 59.62% | 62.58% | 70.59% | 76.90% | 70.86% | 76.90% | 72.86% | 75.00% |
| **Kappa statistic** | 0.2923 | 0.3346 | 0.3096 | 0.3346 | 0.2479 | 0.2773 | 0.2968 | 0.4112 | 0.2930 | 0.4112 | 0.3080 | 0.3902 |
| **S.E.** | 0.0678 | 0.0506 | 0.0707 | 0.0506 | 0.1170 | 0.0737 | 0.0754 | 0.0550 | 0.0761 | 0.0550 | 0.1053 | 0.2081 |
| 1. **Any moderate physical activity in typical week** | (n=161) | (n=338) | (n=156) | (n=338) | (n=52) | (n=155) | (n=153) | (n=328) | (n=150) | (n=328) | (n=69) | (n=20) |
| **Expected Agreement** | 38.56% | 45.96% | 38.45% | 45.96% | 44.23% | 47.70% | 70.41% | 76.42% | 69.95% | 76.42% | 72.13% | 90.00% |
| **Observed Agreement** | 44.72% | 54.14% | 44.23% | 54.14% | 38.91% | 54.19% | 74.51% | 80.79% | 74.00% | 80.79% | 75.36% | 90.00% |
| **Kappa statistic** | 0.1002 | 0.1515 | 0.0940 | 0.1515 | 0.0872 | 0.1242 | 0.1386 | 0.1853 | 0.1349 | 0.1853 | 0.1161 | -- |
| **S.E.** | 0.0495 | 0.0453 | 0.0500 | 0.0453 | 0.0856 | 0.0693 | 0.0799 | 0.0492 | 0.0807 | 0.0492 | 0.1189 | -- |

CATI – Computer Assisted Telephone Interviews; IVR – Interactive Voice Response; CATI🡪IVR indicates IVR as follow up mode (after CATI first contact). IVR🡪CATI indicates CATI as follow up mode (after IVR first contact).

NB: The kappa-statistic measure of agreement is scaled to be 0 when the amount of agreement is what would be expected to be observed by chance; -1 would represent perfect disagreement; and +1 would represent perfect agreement. For intermediate values, Landis and Koch (1977a, 165) suggest the following interpretations: below 0.0 Poor; 0.00 – 0.20 Slight; 0.21 – 0.40 Fair; 0.41 – 0.60 Moderate; **0.61 – 0.80 Substantial, 0.81 – 1.00 Almost perfect. Bolded kappa statistics in the table represent the ‘substantial’, and ‘almost perfect’ agreement categories.**
