# Supplementary material for: Does mobile phone survey method matter? Reliability of computer-assisted telephone interviews and interactive voice response non-communicable diseases risk factor surveys in low and middle income countries
Source: PLoS One. 2019 Apr 10;14(4):e0214450. doi: 10.1371/journal.pone.0214450 (PMC6457489; doi:10.1371/journal.pone.0214450)
Supplement: S3 Table — (DOCX) [file pone.0214450.s003.docx]

S3 Table. Type and length of question and reliability of responses from computer assisted telephone interviews and interactive voice response mobile phone surveys in Bangladesh and Tanzania.

|  |  |  |  | **Bangladesh** | | | | | | **Tanzania** | | | | | |
| --- | --- | --- | --- | --- | --- | --- | --- | --- | --- | --- | --- | --- | --- | --- | --- |
| **Question** | **Type of question** | **Answer Type** | **Expected* Cognitive burden** | **Word count in question (English)** | **Duration of question audio - English(s)** | **Duration of question audio - Bangla(s)** | **IVR Follow up (CATI🡪IVR) (Kappa Statistic)** | **CATI Follow up (IVR🡪CATI) (Kappa Statistic)** | **Difference in Kappa** | **Word count in question (English)** | **Duration of question audio - English(s)** | **Duration of question audio - Swahili(s)** | **IVR Follow up (CATI🡪IVR) (Kappa Statistic)** | **CATI Follow up (IVR🡪CATI) (Kappa Statistic)** | **Difference in kappa** |
|  |  | **MC** | **Low** |  |  |  |  |  |  |  |  |  |  |  |  |
|  |  | **Numeric** | **Medium** |  |  |  |  |  |  |  |  |  |  |  |  |
|  |  | **Y/N** | **High** |  |  |  |  |  |  |  |  |  |  |  |  |
| **Demographics** | |  |  |  |  |  |  |  |  |  |  |  |  |  |  |
| **Age** | Demographic | numeric | Low | 12 | 7 | 9 | 0.439 | 0.609 | -0.170 | 15 | 8 | 10 | 0.547 | 0.682 | -0.135 |
| **Gender** | Demographic | MC | Low | 27 | 12 | 12 | 0.831 | 0.884 | -0.053 | 17 | 13 | 14 | 0.883 | 0.878 | 0.005 |
| **Urban/rural residence** | Demographic | MC | Medium | 40 | 17 | 22 | 0.723 | 0.746 | -0.023 | 45 | 30 | 33 | 0.528 | 0.573 | -0.045 |
| **Schooling** | Demographic | MC | Low | 54 | 30 | 35 | 0.514 | 0.602 | -0.088 | 44 | 27 | 24 | 0.666 | 0.028 | 0.638 |
| **Pearson correlation coefficient (r)** |  |  |  |  |  | 1.000 | -0.167 | -0.389 |  |  |  | 1.000 | -0.375 | -0.449 |  |
|  |  |  |  |  |  |  |  |  |  |  |  |  |  |  |  |
| **NCD Risk Factors** | |  |  |  |  |  |  |  |  |  |  |  |  |  |  |
| **Currently smoking** | Behavior | MC | Low | 27 | 13 | 19 | 0.685 | 0.690 | -0.005 | 32 | 22 | 26 | 0.393 | 0.503 | -0.110 |
| **Alcohol consumption last 30 days** | Behavior | Y/N | Low | 18 | 9 | 9 | 0.640 | 0.538 | 0.102 | 18 | 15 | 15 | 0.657 | 0.763 | -0.106 |
| **Alcohol - 6 or more drinks last 30 days** | Behavior | Y/N | Medium | 74 | 35 | 34 | - | - | - | 44 | 26 | 32 | 0.519 | 0.593 | -0.074 |
| **Fruits in typical week** | Behavior | Y/N | Medium | 26 | 14 | 16 | 0.124 | 0.255 | -0.131 | 18 | 9 | 12 | 0.359 | 0.372 | -0.013 |
| **Vegetables in typical week** | Behavior | Y/N | Medium | 17 | 8 | 10 | 0.495 | 0.168 | 0.327 | 18 | 10 | 13 | 0.077 | 0.396 | -0.319 |
| **Consumption of processed foods high in salt** | Behavior | MC | High | 41 | 22 | 23 | -0.008 | 0.011 | -0.019 | 38 | 30 | 39 | 0.122 | 0.135 | -0.013 |
| **Effort to limit salt** | Behavior | Y/N | Low | 18 | 9 | 10 | 0.074 | 0.247 | -0.173 | 19 | 13 | 14 | 0.081 | 0.218 | -0.137 |
| **Ever been checked blood pressure** | Behavior | Y/N | Low | 23 | 8 | 14 | 0.666 | 0.539 | 0.127 | 23 | 15 | 17 | 0.495 | 0.660 | -0.165 |
| **Ever been diagnosed high blood pressure** | Behavior | Y/N | Low | 24 | 11 | 14 | 0.435 | 0.741 | -0.306 | 28 | 16 | 18 | 0.613 | 0.642 | -0.029 |
| **Ever been checked diabetes** | Behavior | Y/N | Low | 22 | 9 | 16 | 0.652 | 0.540 | 0.112 | 22 | 13 | 15 | 0.479 | 0.675 | -0.196 |
| **Ever been diagnosed diabetes** | Behavior | Y/N | Low | 24 | 10 | 15 | 0.721 | 0.747 | -0.026 | 32 | 19 | 18 | 0.000 | 0.307 | -0.307 |
| **Vigorous physical activity in typical week** | Behavior | Y/N | High | 66 | 40 | 43 | 0.292 | 0.331 | -0.039 | 46 | 29 | 37 | 0.289 | 0.411 | -0.122 |
| **Moderate physical activity in typical week** | Behavior | Y/N | High | 19 | 10 | 10 | 0.100 | 0.154 | -0.054 | 19 | 16 | 18 | 0.139 | 0.185 | -0.046 |
| **Pearson correlation coefficient (r)** |  |  |  |  |  | 1.000 | -0.155 | -0.090 |  |  |  | 1.000 | -0.052 | -0.223 |  |

NB:*Expected cognitive burden is based on authors’ assessments

MC = Multiple choice

Y/N = Yes/No

CATI – Computer Assisted Telephone Interviews; IVR – Interactive Voice Response
