# Supplementary material for: Does mobile phone survey method matter? Reliability of computer-assisted telephone interviews and interactive voice response non-communicable diseases risk factor surveys in low and middle income countries
Source: PLoS One. 2019 Apr 10;14(4):e0214450. doi: 10.1371/journal.pone.0214450 (PMC6457489; doi:10.1371/journal.pone.0214450)
Supplement: S1 File — (PDF) [file pone.0214450.s004.pdf]

2017_02_26 _IVR _Baseline _ Eng V4_Bang V6

Appendix 4: Interactive Voice Response Survey Transcript

SN	Module	English	Bangla	Code	Skipping Options & Other instructions
E_0_1	Error	I am sorry; the response you have entered is not one of the available options. Now I am going to repeat the question for you.	আমি দুঃখিত। আপনি যে উত্তরটি দিয়েছেন তা গ্রহনযোগ্য নয়। প্রশ্নটি আবার করছি।		
E_0_2	Under 18	Sorry. Since you are under 18 years of age, you are not eligible for the survey. Thank you for your time.	দুঃখিত, যেহেতু আপনার বয়স ১৮ বছরের কম, এই জরিপটি আপনার জন্য প্রযোজ্য নয় । সময় দেয়ার জন্য ধন্যবাদ।		
Error_ 0_3	No age	Sorry. Since we could not determine your age, we have to end the survey here. Thank you for your time.	দুঃখিত, যেহেতু,আপনার বয়স সম্পর্কে আমরা নিশ্চিত নই, আমাকে জরিপটি শেষ করতে হচ্ছে। সময় দেয়ার জন্য ধন্যবাদ।		
Error_ 0_4	Refuse	Sorry. Since you are unwilling to participate in the survey, we apologize for any inconvenience. Thank you for your time.	দুঃখিত, যেহেতু আপনি অংশগ্রহন করতে আগ্রহি নন, জরিপটি এখানেই শেষ করছি। সময় দেয়ার জন্য ধন্যবাদ।		
Langu age	Languag e	Hello,<br><br>I am calling from IEDCR which is an institute under the Ministry of Health and Family Welfare of Bangladesh government. This is an automated survey on health. This survey can be taken both in English and Bangla.<br><br>If you would like to listen in Bangla, press 1 or if you would like to listen in English, press 2.	হ্যালো,<br>আমি বাংলাদেশ সরকারের স্বাস্থ্য মন্ত্রণালয়ের অধীনে একটি প্রতিষ্ঠান IEDCR থেকে বলছি ।এটা স্বাস্থ্য বিষয়ক একটি কম্পিউটারাইজড জরিপ। উত্তর দেয়ার জন্য আপনার মোবাইল ফোনের কিপ্যাড অর্থাৎ নাম্বারগুলি ব্যবহার করুন। আপনি যদি বাংলায় শুনতে চান, তাহলে ১ চাপুন, যদি ইংরেজীতে শুনতে চান তাহলে ২ চাপুন।	1 = BANGLA<br>2 = ENGLISH<br>OTHER (Error 0.1)	

M01	Intro-Informational	Thank you. This interview will take a maximum of **30** minutes of your time. All information you provide will be kept confidential, and will only be used to understand the health of the people of our country. To answer each question, I will ask you to press a certain number. At any time, you can repeat the question by pressing the star button.<br><br>If you are unwilling to answer any question, you can press 0. As a token of appreciation for your valuable time, we will provide 50 taka talk time to your phone.<br><br>To listen to the questions clearly and to respond easily, you may turn on your mobile set speaker.	ধন্যবাদ। এই ইন্টারভিউ এর জন্য প্রায় ২৫ মিনিটের মতো সময় লাগবে। আপনার দেয়া সব তথ্য গোপন রাখা হবে, এবং শুধুমাত্র আমাদের দেশের মানুষের স্বাস্থ্য সম্পর্কে বিশদভাবে বোঝার জন্য ব্যবহার করা হবে। মূল্যবান সময় দেয়ার জন্য, আপনার ফোনে ৫০ টাকার টকটাইম পাঠানো হবে। প্রতিটি প্রশ্নের উত্তর দেয়ার জন্য আপনাকে একটি নির্দিষ্ট নাম্বার চাপতে বলা হবে। আগের প্রশ্নটি শুনতে চাইলে স্টার চাপুন। আপনি যদি কোন প্রশ্নের উত্তর দিতে না চান, তাহলে ০ চাপতে পারেন।<br>প্রশ্ন শোনা এবং উত্তর দেয়ার সুবিধার জন্য আপনার মোবাইলের স্পিকার অন করে নিলে ভাল হয়।		
Q1	Consent	If you are ready and willing to take the survey, press 1 or if you do not want to participate, then press 0.	আপনি যদি এই জরিপে অংশ নিতে চান তাহলে, ইন্টারভিউ চালিয়ে যেতে ১ চাপুন। যদি অংশ নিতে না চান, তাহলে ০ চাপুন।	1 = CONTINUE<br>0 = REFUSE<br>OTHER = (E_0_1)	IF 0, READ Error_0_4 (Refuse) and **END SURVEY**
Q2	Demo-	Are you male or female? If you are male, press 1. If you are female, then press 3. You may press 5 in case of other gender.	আপনি পুরুষ হলে, ১ চাপুন। মহিলা হলে, ৩ চাপুন। অন্যান্য ক্ষেত্রে ৫ চাপুন।	1 = MALE<br>3 = FEMALE<br>5 = TRANSGENDER<br>OTHER = (E_0_1)	
Q3 screen	Demo	Are you 18 years or older? If YES, Press 1. If No, Press 3. If you do not want to respond press 0.	আপনি ১৮ বছর বা তার উপরের হলে, ১ চাপুন। আর তার নিচের হলে, ৩ চাপুন। যদি এই প্রশ্নের উত্তর দিতে না চান, তাহলে ০ চাপুন।	1 = >-18<br>3 = 0-17 years old<br>0 = REFUSED<br>OTHER = (E_0_1)	IF 3 OR 0 and READ E_0_2 (UNDER 18 message) and **END SURVEY**
Q3	Demo-	What is your age? To answer this question, use your mobile phone's keypad. For example, if you are 18 years of age, then press 1 and 8. If you do not know your age press 8 for three times.	আপনার বয়স কত? উত্তর দেয়ার জন্য আপনার মোবাইল ফোনের কিপ্যাড অর্থাৎ নাম্বারগুলি ব্যবহার করুন। যেমন, আপনার বয়স যদি ১৮ বছর হয়, প্রথমে ১ তারপর ৮ চাপুন।	**NUM (2), (RANGE 18 - 99)888**= Don't know<br><br>**What is NUM (2)?**	**IF NUM (2), GO TO Q4**<br><br>IF AGE< 18 Repeat Question

			অথবা আপনার বয়স যদি ৩৪ হয় তবে প্রথমে ৩ পরে ৪ চাপুন। যদি আপনার বয়স না জানা থাকে তবে তিন বার ৮ চাপুন।	OTHER=(E_0_1)	
Q4	Demo	Do you live in an urban area, such as, Dhaka, Chittagong or district, or do you live in a rural area? If you live in an urban area, press 1. If you live in a rural area, press 3.	আপনি শহরে না গ্রামে বাস করেন? শহর বলতে বুঝানো হচ্ছে- ঢাকা, চট্টগ্রাম বা যেকোন জেলা-সদর। আপনি যদি শহরে বাস করেন, ১ চাপুন। যদি গ্রামে বাস করেন তাহলে,৩ চাপুন।	1 = Urban<br>3 = Rural<br>0= REFUSED<br>OTHER = (E_0_1)	
Q5	Demo	What is your level of education?<br>If you did not attend school press 1,<br>if completed primary school press 2,<br>if completed SSC or 'O' level press 3,<br>if completed HSC or 'A' level press 4,<br>if completed graduation press 5,<br>if completed post-graduation press 6,<br>if you do not want to respond press 0	আপনি সর্বোচ্চ কতদূর পর্যন্ত পড়াশুনা করেছেন?<br>যদি স্কুলে না যেয়ে থাকেন তাহলে ১ চাপুন,<br>প্রাইমারী স্কুল পাশ করে থাকলে ২ চাপুন,<br>এস.এ.সি অথবা ও লেভেল পাশ করে থাকলে ৩ চাপুন,<br>এইচ.এস.সি অথবা এ লেভেল পাশ করে থাকলে ৪ চাপুন,<br>বিশ্ববিদ্যালয় থেকে স্নাতক অথবা ডিগ্রী নিয়ে থাকলে ৫ চাপুন,<br>স্নাতকোত্তর বা তার চেয়ে বেশি শিক্ষা নিয়ে থাকলে ৬ চাপুন।<br>প্রশ্নের উত্তর দিতে না চাইলে, ০ চাপতে পারেন।	1 = No education<br>2= Primary<br>3 = Secondary school certificate /O level<br>4= Higher Secondary certificate /A level<br>5 = Graduation<br>6 = Post graduation<br>0= Refused	
M02	Tob.	Now, I will ask you about your smoking related activity, such as cigarette, biri and hookah. Please do not report any smokeless tobacco or e-cigarette use. To repeat the question, press the star button and if you do not want to respond, press 0.	আমি এখন আপনাকে সিগারেট, বিড়ি এবং হুক্কার মত ধূমপান সম্পর্কে জিজ্ঞাসা করব। অনুগ্রহ করে ধোয়াবিহীন তামাক অথবা ই-সিগারেট সম্পর্কে উত্তর এখানে উল্লেখ করবেন না। কোন প্রশ্ন আবার শুনতে চাইলে স্টার চাপতে পারেন এবং উত্তর দিতে না চাইলে, ০ চাপতে পারেন।		
Q6	Tob.	Do you currently smoke tobacco? If you smoke every day, press 1. If you smoke occasionally, press 3. If you do not smoke at all, press 5. If you do not want to respond, press 0.	আপনি কি বর্তমানে ধূমপান করেন? যদি প্রতিদিন ধূমপান করেন তাহলে, ১ চাপুন। যদি প্রতিদিন ধূমপান না করেন কিন্তু মাঝেমাঝে ধূমপান করেন তাহলে, ৩ চাপুন। যদি কখনো ধূমপান না করে থাকেন, তাহলে ৫ চাপুন। উত্তর দিতে না	1 = DAILY<br>3 = OCCATIONALY<br>5 = NOT AT ALL<br>0 = REFUSED<br>OTHER = (E_0_1)	If the respondent do not press 1,3,5,0 but press any other button then how many time the question would be

			চাইলে, ০ চাপতে পারেন।		repeated?
M03	Tob.	The following question is about smokeless tobacco, or jorda, shadapata or Gul use. Smokeless tobacco is tobacco that is not smoked but is chewed or placed in the mouth.	পরবর্তী প্রশ্নটি হল ধোয়াবিহীন তামাক, যেমন- জর্দ্দা, সাদাপাতা, গুল সম্পর্কে। ধোয়াবিহীন তামাক হল যা চিবানো হয় অথবা মুখের ভেতর রাখা হয়।		
Q7	Tob.	Do you currently eat Jarda, ShadaPata or use Gul? If you eat Jarda, Shada Pata or use Gul every day, press 1, If you use occasionally, press 3. If you do not use smokeless tobacco at all, press 5. If you do not want to respond, press 0.	আপনি কি জর্দ্দা, সাদাপাতা খান বা গুল ব্যবহার করেন? যদি প্রতিদিন ব্যবহার করেন তাহলে, ১ চাপুন। যদি মাঝেমাঝে ব্যবহার করেন তাহলে, ৩ চাপুন। যদি কখনো ব্যবহার না করেন, তাহলে ৫ চাপুন। প্রশ্নের উত্তর দিতে না চাইলে, ০ চাপতে পারেন।	1 = DAILY<br>3 = OCCATIONALY<br>5 = NOT AT ALL<br>0 = REFUSED<br>OTHER = (E_0_1)	
M04	Tob.	Thank you for answering these questions.	এই প্রশ্নগুলোর উত্তর দেয়ার জন্য আপনাকে ধন্যবাদ।		
M05	Alc.	Now I will ask you about alcohol use. To repeat the question, press the star button or if you do not want to respond, press 0. This survey is confidential, and your answers will not be shared with anyone.	আমি এখন আপনাকে মদ খাওয়া সম্পর্কে প্রশ্ন করব। কোন প্রশ্ন আবার শুনতে চাইলে স্টার চাপতে পারেন এবং কোন প্রশ্নের উত্তর দিতে না চাইলে, ০ চাপতে পারেন। এই জরিপটি গোপনীয় এবং আপনার উত্তর অন্য কারো কাছে প্রকাশ করা হবে না।		
Q8	Alc.	Did you ever in your life drink alcohol such as, beer, wine, spirit, whisky, vodka or Bangla mod? If yes, press 1. If no, press 3. If you do not want to respond, press 0.	আপনি কি কখনো মদ যেমন, বিয়ার, ওয়াইন, স্পিরিট, হুইস্কি ভদকা বা দেশী মদ পান করেছেন? উত্তর হ্যা হলে, ১ চাপুন। না হলে ৩ চাপুন। প্রশ্নের উত্তর দিতে না চাইলে, ০ চাপতে পারেন।	1 = YES<br>3 = NO<br>**0 = REFUSED???**<br>OTHER = (E_0_1)	IF 1 OR 0, GO TO Q9<br>IF 3, GO TO M0.6<br>If the respondent refused to answer this question, can we ask her/him any other question related to Alcohol?(ethical issue)
Q9	Alc.	In the past 30 days, did you drink any alcohol? If yes, press 1. If no, press 3. If you do not want to respond, press 0.	গত একমাসে আপনি কি মদ খেয়েছেন? উত্তর হ্যা হলে, ১ চাপুন। উত্তর না হলে ৩ চাপুন। প্রশ্নের উত্তর দিতে না চাইলে, ০ চাপতে পারেন।	1 = YES<br>3 = NO<br>0 = REFUSED???<br>OTHER = (E_0_1)	IF 1 OR 0, GO TO Q10screen<br>IF 3, GO TO M0.6<br>Same ques.like Q8

Q10 screen	Alc.	Suppose, one drink of alcohol is equal to one can or one and a half regular glass of beer, or a little more than half a glass of wine or one fifth glass of spirit, whisky or Bangla mod. Taking into account all types of alcohol and intoxicating drinks, in the past 30 days; did you ever drink 6 or more such drinks in one sitting? If yes, press 1. If no, press 3.If you do not want to respond, press 0.	মনে করুন, একবার মদ খাওয়া মানে হল এক ক্যান বা একটি সাধারন গ্লাস এর দেড় গ্লাস বিয়ার বা আধা গ্লাস এর চেয়ে কিছু বেশী ওয়াইন বা এক গ্লাস এর পাঁচ ভাগের এক ভাগ স্পিরিট বা হুইস্কি বা দেশী মদ। সব ধরনের মদ বা এলকোহল জাতীয় পানীয় বিবেচনা করে, গত একমাসে আপনি কি কখনো এক বসায় ৬ বার বা তার চেয়ে বেশী মদ খেয়েছেন? হ্যা হলে, ১ চাপুন। না হলে, ৩ চাপুন। প্রশ্নের উত্তর দিতে না চাইলে, ০ চাপতে পারেন।	1 = YES<br>3 = NO<br>0 = REFUSED<br>OTHER = (E_0.1)	IF 1, GO TO Q10<br>IF 0 or 3, GO TO M0.6
Q10	Alcohol	In the past 30 days, how many times did you drink six or more drinks in one sitting? Please enter the number of times this month you drank 6 or more alcoholic drinks in one sitting. E.g. if you drink 4 times this month press 4.	গত একমাসে আপনি কতবার এক বসায় ৬ বার বা তার বেশী মদ খেয়েছেন? যতবার ওই পরিমান মদ খেয়েছেন, অনুগ্রহ করে সেই সংখ্যাটি চাপুন। যেমন - গত একমাসে আপনি যদি চার বার খেয়ে থাকেন তাহলে, ৪ চাপুন।	CHAR(2), RANGE (0-30)<br>OTHER = (E_0_1)	
M06	Alcohol	Thank you for answering these questions.	এই প্রশ্নগুলোর উত্তর দেয়ার জন্য আপনাকে ধন্যবাদ।		
M07	Diet	In the following questions, I will ask you about the types of food you eat. To repeat a question, press the star button.	পরবর্তী প্রশ্নগুলোতে, আপনি যেসব খাবার খান তা সম্পর্কে আপনাকে প্রশ্ন করবো। কোনো প্রশ্ন আবার শুনতে চাইলে স্টার চাপতে পারেন।		
Q11 screen	Diet	Do you eat any fruit e.g. apple, banana, guava, mango, orange or jack fruit in a typical week? If yes, Press 1, if no Press 3. If you do not want to respond, press 0.	আপনি কি একটি সাধারণ সপ্তাহে ফল যেমন, আপেল, কলা, পেয়ারা, আম, কমলা, কাঁঠাল খান ? যদি হ্যা হয়, ১ চাপুন। আর না হলে, ৩ চাপুন। প্রশ্নের উত্তর দিতে না চাইলে ০ চাপতে পারেন।	1 = YES<br>3 = NO<br>0 = REFUSED<br>OTHER = (E_0_1)	If 3 OR 0 GO TO Q13 screen
Q11	Diet	In a typical week, how many days do you eat fruits? Please enter the number of days. If you do not want to respond, press 0.	একটি সাধারণ সপ্তাহে আপনি কত দিন ফল খান? অনুগ্রহ করে দিনের সংখ্যা চাপুন। আপনি এই প্রশ্নের উত্তর দিতে না চাইলে ০ চাপতে পারেন।	MC, RANGE (01-7)<br>0 = REFUSED<br>OTHER = (E_0_1)	IF 1-7 GO TO Q12<br>IF 0, GO TO Q13screen

Q12	Diet	Suppose, one serving of fruit is a medium apple, banana, guava, mango or orange or jack fruit; or half a cup of chopped fruit. How many of such servings of fruits do you eat in one of those days? Please press the serving number. If you do not want to respond, press 0.	মনে করুন, এক পরিবেশন ফল মানে হল, একটি মাঝারি আকৃতির আপেল, কলা, পেয়ারা, আম, কমলা বা কাঁঠাল অথবা আধা কাপ কাটা ফল। আপনি যে দিনগুলোতে ফল খান, এমন একটি দিনে এধরনের কত পরিবেশন ফল খান? অনুগ্রহ করে পরিবেশনের সংখ্যাটি চাপুন। যেমন - এক দিনে যদি এধরনের চার পরিবেশন ফল খেয়ে থাকেন তাহলে, ৪ চাপুন। আপনি এই প্রশ্নের উত্তর দিতে না চাইলে ০ চাপতে পারেন।	MC, RANGE (1– 9)<br>0 = REFUSED<br>OTHER = (E_0_1)	
Q13 screen	Diet	Do you eat any vegetables in a typical week? If yes Press 1, if no Press 3. If you do not want to respond, press 0.	আপনি কি একটি সাধারণ সপ্তাহে শাক-সব্জী খান? যদি হ্যা হয়, ১ চাপুন। আর না হলে, ৩ চাপুন। যদি উত্তর দিতে না চান, তাহলে ০ চাপুন।	1 = YES<br>3 = NO<br>0 = REFUSED<br>OTHER = (E_0.1)	If 3 OR 0 GO TO M0.8
Q13	Diet	On a typical week, how many days do you eat vegetables or green leafs? Please enter the number of days. If you do not want to respond, press 0.	একটি সাধারণ সপ্তাহে আপনি কত দিন শাক-সব্জী খান? অনুগ্রহ করে দিনের সংখ্যাটি চাপুন। আপনি যদি এই প্রশ্নের উত্তর দিতে না চান তাহলে, ০ চাপতে পারেন।	MC, RANGE (1-7)<br>0 = REFUSED<br>OTHER = (E_0_1)	IF 1-7 GO TO Q14<br>IF 0, GO TO M0.8
Q14	Diet	One serving means a cup of vegetable or green leafs or salad; or half a cup of cooked vegetables or green leafs. How many of these servings of vegetables do you eat in one of those days? Please enter the number of servings. For example, if you eat 5 servings of vegetables in one of those days, Press 5. If you do not want to respond, press 0. To repeat a question, press the star button	এক পরিবেশন মানে হল প্রায় এক কাপ সবুজ শাক-সব্জী, সালাদ অথবা আধা কাপ রান্না করা শাক-সব্জী । আপনি যে দিনগুলোতে শাক-সব্জী খান, এমন একদিনে এধরনের কত পরিবেশন শাক-সব্জী খান? অনুগ্রহ করে পরিবেশনের সংখ্যাটি চাপুন। যেমন - একদিনে এধরনের পাঁচ পরিবেশন শাক-সব্জী খেয়ে থাকেন তাহলে, ৫ চাপুন। আপনি এই প্রশ্নের উত্তর দিতে না চাইলে ০ চাপতে পারেন।প্রশ্নটি আবার শুনতে চাইলে স্টার চাপুন।	MC, RANGE (1-9)<br>0 = REFUSED<br>OTHER = (Error 0_1)	
M08	Diet	In the following questions, I would like to ask you about your salt intake. Think about all sources of salt, such as table salt, salted stock cubes and spices, soy sauce, fish sauce etc. To repeat a question,	পরবর্তী প্রশ্নগুলোতে, আপনার লবণ খাওয়ার অভ্যাস সম্পর্কে জিজ্ঞাসা করবো। আপনি যেকোন লবণ, যেমন - সাধারণ খাবার লবণ, লবণযুক্ত স্টককিউব ও মশলা, সয়াসস, মাছের		

		press the star button. If you do not want to respond, press 0.	সস, এধরনের সব রকম লবণের উৎস সম্পর্কে চিন্তা করুন। আপনি কোনো প্রশ্ন আবার শুনতে চাইলে স্টার চাপতে পারেন এবং কোন প্রশ্নের উত্তর দিতে না চাইলে ০ চাপতে পারেন।		
Q15	Diet<br><br>Salt in cooking	During cooking or preparing food in your household, how many times do you use salt, If you use every time, press 1. If you use occasionally, press 3. If you do not use at all, press 5. If you do not know, press 7. If you do not want to respond, press 0.	আপনার ঘরে রান্না করার সময় কতবার লবণ ব্যবহার করা হয়? যদি সবসময় ব্যবহার করা হয়, তাহলে, ১ চাপুন। যদি মাঝেমাঝে ব্যবহার করা হয় তাহলে, ৩ চাপুন। যদি কখনোই ব্যবহার না করা হয় তাহলে, ৫ চাপুন। যদি না জানেন তাহলে, ৭ চাপুন। প্রশ্নের উত্তর দিতে না চাইলে ০ চাপতে পারেন।	1 = ALWAYS<br>3 = OCCASSIONALLY<br>5 = NEVER<br>7= DON'T KNOW<br>0 = REFUSED<br>OTHER = (E_0.1)	
Q16	Diet<br>Salty sauce in cooking	During cooking or preparing food in your household, how many times do you use salty sauce, salty pickles, chutney in your dishes? If you use every time, press 1. If you use occasionally, press 3. If you do not use at all, press 5. If you do not know, press 7. If you do not want to respond, press 0.	আপনার বাড়িতে রান্না করার সময় লবণ যুক্ত মশলা, সস, ইত্যাদি ব্যবহার করা হয় কি? যদি সবসময় ব্যবহার করা হয়, তাহলে, ১ চাপুন। যদি মাঝেমাঝে ব্যবহার করা হয় তাহলে, ৩ চাপুন। যদি কখনোই ব্যবহার না করা হয় তাহলে, ৫ চাপুন। যদি না জানেন তাহলে, ৭ চাপুন। প্রশ্নের উত্তর দিতে না চাইলে ০ চাপতে পারেন।	1 = ALWAYS<br>3 = OCCASSIONALLY<br>5 = NEVER<br>7= DON'T KNOW<br>0 = REFUSED<br>OTHER = (E_0.1)	
Q17	Diet<br><br>Use of extra salt in table	When you eat, do you add extra salt? If you use every time, press 1, if sometimes, press 3, if you do not use at all, press 5. If you do not want to respond, press 0.	আপনি কি খাবার খাওয়ার সময় পাতে বাড়তি লবন খান? যদি সব সময় খান তাহলে, ১ চাপুন। যদি মাঝেমাঝে খান তাহলে, ৩ চাপুন। যদি কখনোই না খান তাহলে, ৫ চাপুন। প্রশ্নের উত্তর না দিতে চাইলে ০ চাপতে পারেন।	1 = ALWAYS<br>3 = SOMETIMES<br>5 = NEVER<br>0 = REFUSED<br>OTHER = (E_0.1)	
Q18	Diet<br><br>Useof salty sauce in table	When you are eating, do you add salty sauces, pickles or chutney in your dishes? If you use every time, press 1. If you use occasionally, press 3. If you do not use at all, press 5.If you do no t want to respond, press 0.	আপনি কি খাবার খাওয়ার সময় লবণযুক্ত সস, আঁচার, চাটনি ইত্যাদি খান? যদি সব সময় খান তাহলে, ১ চাপুন। । যদি মাঝেমাঝে খান তাহলে, ৩ চাপুন। যদি কখনোই না খান তাহলে, ৫ চাপুন। প্রশ্নের উত্তর না দিতে চাইলে ০ চাপতে পারেন।	1 = ALWAYS<br>3 = OCCASSIONALLY<br>5 = NEVER<br>7= DON'T KNOW<br>0 = REFUSED<br>OTHER = (E_0.1)	

Q19	Diet<br><br>Salty snakes	Typically, how many times do you eat packaged salty snacks such as cheese, biscuit, chips, or, processed meats and fish? If you eat always, press 1. If you eat occasionally press 3. If you do not eat at all, press 5.If you do not want to respond, press 0.	আপনি সাধারনত, কত ঘনঘন লবণযুক্ত প্যাকেটের খাবার যেমন, পনির, বিস্কুট, চিপ্স, অথবা টিনের প্রক্রিয়াজাত মাছ/মাংস খান? যদি সবসময় খান তাহলে ১ চাপুন। যদি মাঝেমাঝে খান তাহলে, ৩ চাপুন। যদি কখনোই না খান তাহলে, ৫ চাপুন। প্রশ্নের উত্তর না দিতে চাইলে ০ চাপতে পারেন।	1 = ALWAYS<br>3 = OCCASSIONALLY<br>5 = NEVER<br>0 = REFUSED<br>OTHER = (E_0.1)	
Q 20	Diet<br>Salt restriction	Currently, are you doing anything to limit your salt intake? If yes, press 1. If no, press 3. If you do not want to respond, press 0.	আপনি কি এখন লবণ খাওয়া কমানোর চেষ্টা করছেন? যদি হ্যাঁ হয়, তাহলে ১ চাপুন। যদি না হয় তাহলে ৩ চাপুন। প্রশ্নের উত্তর দিতে না চাইলে ০ চাপতে পারেন।	1 = YES<br>3 = NO<br>0 = REFUSED<br>OTHER = (E_0_1)	
M09	Diet	Thank you for answering these questions	প্রশ্নগুলোর উত্তর দেয়ার জন্য আপনাকে ধন্যবাদ।		
M10	BP/Dia	Now I will ask you questions about your physical conditions, treatments, and use of medicines. to repeat a question press the star button. If you do not want to respond, press 0.	এখন আপনার শারিরীক অবস্থা, চিকিৎসা এবং ঔষধ খাওয়া সম্পর্কে প্রশ্ন করবো। কোনো প্রশ্ন আবার শুনতে চাইলে স্টার চাপতে পারেন এবং কোন প্রশ্নের উত্তর দিতে না চাইলে ০ চাপতে পারেন।		
Q 21	BP/Dia	If you ever had your blood pressure measured by a doctor or health care worker, did they ever diagnose you with high blood pressure? If yes, press 1. If no, press 3. If you have never had your blood pressure measured, press 5. If you do not want to respond, press 0.	আপনাকে কখনো কোন ডাক্তার বা স্বাস্থ্যকর্মী কি জানিয়েছেন যে,আপনার উচ্চ রক্তচাপ বা হাই ব্লাড প্রেশার আছে? যদি উত্তর হ্যা হয় তাহলে, ১ চাপুন। যদি না হয় তাহলে ৩ চাপুন। যদি কখনো রক্তচাপ বা ব্লাড প্রেশার পরীক্ষা না করিয়ে থাকেন তাহলে, ৫ চাপুন। প্রশ্নের উত্তর না দিতে চাইলে ০ চাপতে পারেন।	1 = YES<br>3 = NO<br>5 = NEVER HAD BP TAKEN<br>0 = REFUSED<br>OTHER = (E_0_1)	IF 1 GO TO Q22<br>IF 3 OR 5 OR 0, GO TO Q23
Q22	BP/Dia	In the past two weeks, did you take any medicine or diet advice for high blood pressure prescribed by a doctor or health care worker? If yes, press 1. If no, press 3. If you do not want to respond, press 0.	গত দুই সপ্তাহে,আপনি কি কখনো উচ্চ রক্তচাপ বা হাই ব্লাড প্রেশারের জন্য ডাক্তার বা স্বাস্থ্যকর্মীর দেয়া কোন ঔষধ খেয়েছেন কিংবা খাবারের ব্যাপারে কোন উপদেশ মেনে চলছেন? যদি হ্যা হয় তাহলে, ১ চাপুন। যদি না হয় তাহলে,	1 = YES<br>3 = NO<br>0 = REFUSED<br>OTHER = (E_0_1)	

			৩ চাপুন। প্রশ্নের উত্তর না দিতে চাইলে ০ চাপতে পারেন।		
Q23	BP/Dia	Has any doctor or any health care worker ever diagnosed you with high blood sugar or diabetes? If yes, press 1. If no, press 3. If you do not want to respond, press 0.	আপনাকে ডাক্তার অথবা কোন স্বাস্থ্যকর্মী কি জানিয়েছেন যে,আপনার রক্তে সুগার বেশী আছে অথবা আপনার ডায়াবেটিস আছে। যদি উত্তর হ্যা হয় তাহলে, ১ চাপুন। যদি না হয় তাহলে, ৩ চাপুন। প্রশ্নের উত্তর না দিতে চাইলে ০ চাপতে পারেন।	1 = YES<br>3 = NO<br>0 = REFUSED<br>OTHER = (E_0_1)	IF 1 GO TO Q24<br>IF 3 OR 0, GO TO M1_1
Q24	BP/Dia	In the past two weeks, did you take any medicine or insulin, prescribed by a doctor or a health care worker? If yes, press 1. If no, press 3.If you do not want to respond, press 0.	গত দুই সপ্তাহে,আপনি কি ডায়াবেটিসের জন্য ডাক্তার বা স্বাস্থ্যকর্মীর দেয়া কোন ঔষধ খেয়েছেন কিংবা ইনসুলিন নিয়েছেন? যদি উত্তর হ্যা হয় তাহলে, ১ চাপুন। যদি না হয় তাহলে, ৩ চাপুন। প্রশ্নের উত্তর না দিতে চাইলে ০ চাপতে পারেন।	1 = YES<br>3 = NO<br>0 = REFUSED<br>OTHER = (E_0_1)	
M11	BP/Dia	Thank you for answering these questions.	এই প্রশ্নগুলোর উত্তর দেয়ার জন্য আপনাকে ধন্যবাদ।		
M12	GPAQ	Now I am going to ask you the time you spend being physically active in a typical week. Count the duration of how long you are physically active at work, at home at least for 10 minutes. First, I will ask you about your vigorous physical activity and then about moderate physical activity. To repeat a question, press the star button.	একটি সাধারণ সপ্তাহে আপনি কতক্ষন শারিরীক পরিশ্রম করেন তা আমি এখন জানতে চাচ্ছি।একটু চিন্তা করুন যে, আপনি কর্মক্ষেত্র, বা বাড়িতে কিংবা অন্য সময়েকমপক্ষে দশ মিনিটের বেশী কতটুকু সময় পরিশ্রম করছেন ।আমি প্রথমে অতিরিক্ত পরিশ্রমের কাজ এবং পরে মাঝারি পরিশ্রমের কাজ নিয়ে আলাপ করব। আপনি কোন প্রশ্ন আবার শুনতে চাইলে স্টার চাপতে পারেন।		
Q25screeb	GPAQ (vigorous-intensity activity)	In a typical week, do you do any vigorous intensity activity? Which means activity (at least for ten minutes) that causes large increase in breathing or heart rate; like carrying heavy loads, digging earth, construction work, fast bicycling, swimming, pulling a rickshaw, heavy household chores like, washing clothes, cleaning floor and grinding spices using a	একটি সাধারন সপ্তাহে, আপনি কি কঠোর শারিরীক পরিশ্রম করেন? কঠোর শারিরীক পরিশ্রম বলতে বুঝানো হচ্ছে যে, আপনি কমপক্ষে দশ মিনিট এমন কোন শারিরীক পরিশ্রম বা ব্যায়াম করেন যার জন্য ঘন ঘন নিঃশ্বাস নিতে হয় অথবা হৃদস্পন্দন বেড়ে যায় । যেমন, ভারী জিনিস তোলা / বহণ	1 = YES<br>3 = NO<br>0 = REFUSED<br>OTHER = (E_0_1)	If 3 OR 0, GO TO M1.3 (IPAQ)

		local grinder etc.<br><br>If Yes, Press 1. If No, press 3.If you do not want to respond, press 0.	করা, মাটিকাটা, নির্মানকাজ, দ্রুত সাইকেল চালানো, সাতারকাটা, রিকশা চালানো, গৃহস্থালীর ভারী কাজ যেমন, কাপড় ধোঁয়া, ঘর মোছা, মশলা বাটা, ইত্যাদি। উত্তর যদি হ্যাঁ হয়, ১ চাপুন, না হলে ৩ চাপুন। আপনি এই প্রশ্নের উত্তর দিতে না চাইলে ০ চাপতে পারেন।		
Q25	GPAQ (vigorous -intensity activity)	In a typical week, how many days do you do vigorous intensity activity? Please press the number of days. If you do not want to respond press 0.	একটি সাধারন সপ্তাহে, আপনি কতদিন কঠোর শারিরীক পরিশ্রম করেন? অনুগ্রহ করে দিনের সংখ্যাটি চাপুন। আপনি এই প্রশ্নের উত্তর দিতে না চাইলে ০ চাপতে পারেন।	MC, RANGE (1-7)<br>0 = REFUSED<br>OTHER = (E_0_1)	IF 1-7 GO TO Q26<br>IF 0, GO TO M1.3 (IPAQ)
Q26	GPAQ (vigorous -intensity activity)	On one of those days, how many hours do you do vigorous intensity activity? If you do for one hour or less, press 1. If you do more than 1 hour press 3. If you do not want to respond, press 0.	এইরকম একটি দিনে আপনি সাধারনত কতক্ষণ এধরনের কঠোর শারিরীক পরিশ্রম বা ব্যায়াম করেন? ১ ঘন্টা বা তারচেয়ে কম হলে, ১ চাপুন; ১ ঘন্টার বেশী হলে ৩ চাপুন। আপনি এই প্রশ্নের উত্তর দিতে না চাইলে ০ চাপতে পারেন।	1 = ≤1 hour<br>3 = > 1 hour<br>0 = REFUSED<br>OTHER = (E_0_1)	
M13	GPAQ (moderat e _intensity activity)	In a typical week, think about your moderate intensity activity. Moderate intensity activity (at least for 10 minutes) that causes small increase in breathing or heart rate e.g. carrying light loads, rowing a boat or riding a bicycle with a regular pace. Do not count walking in this activity.	একটি সাধারন সপ্তাহে, আপনার মাঝারি মানের শারিরীক পরিশ্রমের কথা চিন্তা করুন। মাঝারি মানের শারিরীক পরিশ্রম বলতে বোঝানো হচ্ছে যে, আপনি কমপক্ষে দশ মিনিট এমন কোন শারিরীক পরিশ্রম বা ব্যায়াম করেন যার জন্য আপনাকে স্বাভাবিকের চাইতে একটু বেশী শ্বাঃস নিতে হয় অথবা হৃদস্পন্দন বেড়ে যায় এবং সেটা হতে পারে হালকা জিনিস বহন করা, স্বাভাবিক গতিতে সাইকেল চালানো, নৌকা চালানো, ইত্যাদি। হাঁটাকে এখানে উল্লেখ করবেন না।		
Q27 Screen	GPAQ<br><br>(moderat e _intensity activity)	In a typical week, do you do any moderate intensity activity? If Yes, Press 1. If No, press 3. If you do not want to respond, press 0.	একটি সাধারন সপ্তাহে, আপনি কি এরকম মাঝারি মানের শারিরীক পরিশ্রম করেন? যদি হ্যাঁ হয়, ১ চাপুন. আর না হলে ৩ চাপুন। উত্তর দিতে না চাইলে ০ চাপতে পারেন।	1 = YES<br>3 = NO<br>0 = REFUSED<br>OTHER = (E_0_1)	IF 3 OR 0, go to M1.4 (IPAQ)

Q27	GPAQ (moderate _intensity activity)	In a typical week, on how many days do you do moderate intensity activity? Please press the number of days. If you do not want to respond, press 0.	একটি সাধারন সপ্তাহে, আপনি কতদিন এধরনের মাঝারি মানের শারিরীক পরিশ্রম করেন? অনুগ্রহ করে দিনের সংখ্যাটি চাপুন। উত্তর দিতে না চাইলে ০ চাপতে পারেন।	MC RANGE (1-7)<br>0 = REFUSED<br>OTHER = (E_0_1)	IF 1-7 GO TO Q28<br>IF 0, GO TO M1.4 (IPAQ)
Q28	GPAQ (moderate _intensity activity)	In a typical day, how many hours do you do moderate intensity activity? If you do for one hour or less press 1. If you do more than 1 hour press 3. If you do not want to respond, press 0..	সাধারনত এরকম একটি দিনে আপনি কতঘন্টা এধরনের মাঝারি মানের শারিরীক পরিশ্রম করেন? এক ঘন্টা বা তারচেয়ে কম হলে, ১ চাপুন; এক ঘন্টার বেশী হলে ৩ চাপুন। আপনি এই প্রশ্নের উত্তর দিতে না চাইলে ০ চাপতে পারেন।	1 = ≤1 hour<br>3 = > 1 hour<br>0 = REFUSED<br>OTHER = (E_0_1)	
M14	GPAQ Walk	Now think about how long you walk on a typical week. Count the time you walk at work and at home, your walk from one place to another.	এখন আপনার হাঁটার অভ্যাস সম্পর্কে জানতে চাইবো। এটা হলো, এক জায়গা থেকে আরেক জায়গায় যাওয়ার জন্য হাঁটা , হেটে কাজে যাওয়া , হেটে বাজারে যাওয়া, হেঁটে মসজিদে যাওয়া, ঘরের ভিতরে হাঁটা, অবসর সময় হাঁটা,ইত্যাদি।		
Q29 Screen	GPAQ Walk	In a typical week, do you walk for at least 10 minutes continuously? If Yes, Press 1. If No, press 3. If you do not want to respond, press 0.	একটি সাধারন সপ্তাহে, আপনি কি অন্তত ১০ মিনিট এইরকম হাঁটেন? যদি হ্যাঁ হয়, ১ চাপুন. আর না হলে ৩ চাপুন। উত্তর দিতে না চাইলে ০ চাপতে পারেন।	1 = YES<br>3 = NO<br>0 = REFUSED<br>OTHER = (E_0_1)	IF 3 OR 0, go to M1.5 (IPAQ)
Q29	GPAQ walk	In a typical week, how many days do you walk for at least 10 minutes continuously? Please enter the number of days. If you do not want to respond, press 0.	একটি সাধারন সপ্তাহে, আপনি কতদিন ঘরের ভিতরে,কাজের যায়গায় বা অন্য যে কোনো প্রয়োজনে কমপক্ষে দশ মিনিটের জন্য হাঁটেন? অনুগ্রহ করে এইরকম দিনের সংখ্যাটি চাপুন ।আপনি এই প্রশ্নের উত্তর দিতে না চাইলে ০ চাপতে পারেন।	MC, RANGE (1-7)<br>0 = REFUSED<br>OTHER = (E_0_1)	IF 1-7 GO TO Q30<br>IF 0, GO TO M1.5 (IPAQ)
Q30	GPAQ Walk	In a typical day how much time do you spend walking? If you walk for one hour or less, press 1. If you walk more than 1 hour press 3. If you do not want to answer, press 0.	এরকম একটি দিনে আপনি সাধারনত কতক্ষণ হাটেন? এক ঘন্টা বা তারচেয়ে কম হলে, ১ চাপুন; এক ঘন্টার বেশী হলে ৩ চাপুন। আপনি এই প্রশ্নের উত্তর দিতে না চাইলে ০ চাপতে পারেন।	1 = ≤1 hour<br>3 = > 1 hour<br>0 = REFUSED<br>OTHER = (E_0_1)	

M15	GPAQ	Thank you for answering these questions	প্রশ্নগুলোর উত্তর দেয়ার জন্য আপনাকে ধন্যবাদ।		
M16	Thank You	This survey is now finished. Thank you for your time. As a token of appreciation, we will provide you with 50 taka talk time to your phone.	জরিপটি এখানেই শেষ । সময় দেয়ার জন্য আপনাকে ধন্যবাদ। মূল্যবান সময় দেয়ার জন্য আপনার ফোনে ৫০ টাকার টক টাইম পাঠানো হয়েছে।		

Revised on 26th February 2017 (only some spelling mistakes were corrected)
