# Supplementary material for: Does mobile phone survey method matter? Reliability of computer-assisted telephone interviews and interactive voice response non-communicable diseases risk factor surveys in low and middle income countries
Source: PLoS One. 2019 Apr 10;14(4):e0214450. doi: 10.1371/journal.pone.0214450 (PMC6457489; doi:10.1371/journal.pone.0214450)
Supplement: S2 File — (PDF) [file pone.0214450.s005.pdf]

MICROTRIAL 5- FULL IVR SURVEY

SN	Module	Question Type	Question Wording	Response Options	Skip
Error _0_1	Error	MSG	I'm sorry, the response you have entered is not one of the available options. I'm now going to repeat the question for you.<br>**Samahani, Jibu ulilotoa halikukubaliwa. Niruhusu nirudie swali.**		
Error _0_2	Under 18	MSG	I'm sorry, since you are under 18 years of age, you are not eligible for this survey. Thank you for your time.<br>**Samahani, kwa kuwa umri wako ni chini ya miaka 18 huwezi kushiriki katika utafiti huu. Asante kwa muda wako.**		
Error _0_3	No age	MSG	I'm sorry, since we cannot determine your age, I will have to end this survey. Thank you for your time.<br>**Samahani, kwa kuwa hatuwezi kubainisha umri wako, nitasitishia hapa utafiti huu. Asante kwa muda wako.**		
Error _0_4	Refuse	MSG	I'm sorry that you do not want to participate and apologize for any inconvenience. Thank you for your time.<br>**Samahani kwa usumbufu wowote kama hautaki kushiriki. Asante kwa muda wako.**		
Language	Demo	MC	Habari,Huu ni utafiti wa afya kutoka Taasisi ya afya Ifakara.Utafiti huu unaweza kufanyika kwa Kiswahili au Kiingereza.<br>Kama unataka mahojiano kwa Kiswahili bonyeza 1<br>If you would like to listen in English Press 2	1 = Swahili<br>2 = English<br>OTHER = (Error_0_1)	
M0.1	Demo	MSG	**[INSERT APPROPRIATE INTRO BASED ON MICRO TRIAL]**		
Q1screen	Demo	MC	Are you 18 years or older? If YES, Press 1. If No, Press 3<br>**Je, una umri wa miaka 18 au zaidi? Kama NDIYO Bonyeza 1. Kama HAPANA Bonyeza 3**	1 = ≥18<br>3 = 0-17 years old<br>0 = REFUSED<br>OTHER = (Error_0_1)	IF 3 OR 0, END SURVEY and READ Error_0_2
Q1	Demo	NUM	How old are you? Use the number buttons on your phone to enter your age.<br>**Una umri wa miaka mingapi? Tumia vitufe vya namba katika simu yako kuingiza umri wako.**	NUM (2), (RANGE 18 - 99)<br>OTHER = (Error_0_1)	IF AGE≥18, GO TO Q3<br>IF AGE< 18 Repeat Question
Q2	Demo	MC	Congratulations, YOU QUALIFY TO TAKE THIS SURVEY, IF YOU WOULD LIKE TO CONTINUE, press 1. If you do not want to participate in this survey at this time, press 0.<br>**Hongera, unastahili kuendelea na utafiti huu, Kama uko tayari**	1 = CONTINUE<br>0= REFUSE<br>OTHER = (Error_0_1)	IF 0, READ (Error_0_4) and END SURVEY

			**kuendelea bonyeza 1. Kama hutaki kushiriki katika utafiti huu kwa muda huu bonyeza 0**		
Q3	Demo	MC	Are you MALE or FEMALE? If you are male, press 1. If you are female, press 3.<br>**Je wewe ni mwanaume au mwanamke? Kama ni mwanaume, bonyeza 1. Kama ni mwanamke, bonyeza 3.**	1 = MALE<br>3 = FEMALE<br>OTHER = (Error_0_1)	
Q4	Demo	MC	Do you live in a city or town? for example, a city like Dar, Mwanza, Arusha, Mbeya, Tanga or do you live in a rural area? If you live in a city or town, press 1. If you live in a rural area, press 3.<br>**Unaishi katika jiji au mji, kwa mfano, jiji kama (Dar, Mwanza, Arusha, Mbeya, Tanga) au unaishi katika eneo la vijiji? Kama unaishi katika jiji au mji, bonyeza 1, kama unaishi katika eneo la vijiji, bonyeza 3**	1 = URBAN<br>3 = RURAL<br>0= REFUSED<br>OTHER = (Error_0_1)	
Q5	Demo	NUM	What is the highest level of education you attended? Press 1 if you didn't go to school. Press 3 if you only attended primary school. Press 5 if you only attended secondary education. Press 7 if you attended any tertiary education or higher education.<br>**Ni kiwango gani cha elimu ulichokomea? bonyeza 1 Kama hukusoma shule, Bonyeza 3 kama ulisoma shule ya msingi, bonyeza 5 kama ulisoma elimu ya sekondari, na bonyeza 7 kama ni Elimu ya Juu**	1 = NO SCHOOL<br>3 = PRIMARY<br>5 = SECONDARY<br>7 = TERTIARY<br>0 = REFUSED<br>OTHER = (Error_0_1)	
M0.2	Tob.	MSG	I would now like to ask you about smoking tobacco,including cigarettes,cigars,shisha and pipes. Please do not include smokeless tobacco such as ugoro. As a reminder, you can press * if you'd like to have a question repeated and you can press 0 if you'd like to skip a question.<br>**Ningependa sasa kukuuliza kuhusu uvutaji tumbaku kama sigara,cigars, shisha. Tafadhali usijumuishe uvutaji wa tumbaku isiyo na moshi kwa wakati huu kama ugoro. Kumbuka kubonyeza nyota kwa swali unalotaka lirudiwe. Na unaweza kubonyeza sifuri kuruka swali lolote ambalo usingependa kujibu**		
Q6	Tob.	MC	Do you currently SMOKE TOBACCO? If you smoke tobacco DAILY, press 1. If you smoke tobacco sometimes, but NOT EVERY DAY, press 3. If you don't smoke tobacco AT ALL, press 5.<br>**Je,UNAVUTA TUMBAKU/ sigara kwa sasa? Kama unavuta KILA SIKU, bonyeza 1. Kama kuna wakati unavuta sigara yenye tumbaku, lakini SIO KILA SIKU, bonyeza 3, kama huvuti KABISA, bonyeza 5.**	1 = DAILY<br>3 = NOT EVERY DAY<br>5 = NOT AT ALL<br>0 = REFUSED<br>OTHER = (Error_0_1)	
M0.3	Tob.	MSG	The next question is about using smokeless tobacco including ugoro. Smokeless tobacco is tobacco that is not smoked but it is either chewed/sniffed.<br>**Swali linalofuata ni kuhusu kutumia tumbaku isiyo na moshi kama**		

			**ugoro. Tumbaku isiyo na moshi ni tumbaku ambayo haivutwi lakini inanuswa kupitia puani, kushikiliwa mdomoni au kutafunwa.**		
Q7	Tob.	MC	Do you currently use SMOKELESS TOBACCO? If you use smokeless tobacco DAILY, press 1. If you use smokeless tobacco sometimes, but NOT EVERY DAY, press 3. If you do not use smokeless tobacco AT ALL, press 5.<br>**Kwa sasa unatumia TUMBAKU ISIYO NA MOSHI? kama unatumia tumbaku isiyo na moshi kwa KILA SIKU, bonyeza 1. Kama kuna wakati huwa unatumia tumbaku isiyo na moshi lakini SIYO KILA SIKU, bonyeza 3. Kama hutumii tumbaku isiyo na moshi KABISA, bonyeza 5**	1 = DAILY<br>3 = NOT EVERY DAY<br>5= NOT AT ALL<br>0 = REFUSED<br>OTHER = (Error_0_1)	
M0.4	Tob.	MSG	Thank you for answering these questions.<br>**Asante kwa kujibu maswali haya.**		
M0.5	Alcl	MSG	Now, I will ask you questions about alcohol. As a reminder you can press the * key for any question that you need to have repeated and you can press zero to skip any question you prefer not to answer. This survey is confidential and your responses will not be shared.<br>**Sasa, nitakuuliza maswali kuhusu pombe. Kama kukukumbusha unaweza kubonyeza kitufe cha nyota kwa swali lolote unalotaka lirudiwe na unaweza kubonyeza sifuri kuruka swali usilopendelea kulijibu. Utafiti huu ni wa siri na majibu yako hayatashirikishwa kwa mtu yeyote asiyehusika.**		
Q8	Alcl	MC	Have you EVER drunk any alcohol such as beer, wine, spirits or local brews? If YES, press 1. If NO, press 3.<br>**Ulishawahi kunywa pombe yeyote kama <u>bia, mvinyo, pombe kali, viroba, au pombe ya kienyeji</u>? kama NDIO bonyeza 1, kama HAPANA bonyeza 3**	1 = YES<br>3 = NO<br>0 = REFUSED<br>OTHER = (Error_0_1)	IF 1, GO TO Q9<br>IF 3 OR 0, GO TO M0.6
Q9	Alcl	MC	During the last 30 days, did you drink **ANY** alcohol? If YES, press 1. If NO, press 3.<br>**Kwa kipindi cha siku 30 zilizopita ulikunywa pombe YEYOTE? Kama NDIO bonyeza 1, kama HAPANA bonyeza 3.**	1 = YES<br>3 = NO<br>0 = REFUSED<br>OTHER = (Error_0_1)	IF 1, GO TO Q10screen<br>IF 3 OR 0, GO TO M0.6
Q10_screen	Allc	MC	One drink is a bottle of beer,a glass of wine or shot of liquor, keeping in mind all alcoholic beverages, have you drank six or more alcoholic beverages in one instance in the past 30 days? Press 1 if Yes, Press 3 if No?<br>**Kinywaji kimoja ni bia moja,glasi moja ya mvinyo au glass ndogo ya pombe kali. Ulishawahi kunywa 6 au Zaidi katika tukio la unywaji wa mara moja katika kipindi cha siku 30 zilizopita? Bonyeza 1 kama NDIO, Bonyeza 3 kama HAPANA**	1 = YES<br>3 = NO<br>0 = REFUSED<br>OTHER = (Error_0_1)	IF 1, GO TO Q10<br>IF 0 or 3, GO TO M0.6

Q10	Alc	NUM	How many times during the **last 30 days** did you have 6 or more drinks in a **single** drinking occasion? Please enter the number of times in the past 30 days that you had 6 or more drinks in one occasion.<br>**Ni mara ngapi katika kipindi cha siku 30 zilizopita Ulipata vinywaji 6 au Zaidi katika tukio moja la kunywa? Tafadhali ingiza namba ya idadi katika katika siku 30 zilizopita ulizokunywa vinyaji 6 au Zaidi katika tukio moja.**	CHAR(2), RANGE (0-30)<br>OTHER = (Error_0_1)	
M0.6	Alc	MSG	Thank you for answering these questions<br>**Asante kwa kujibu maswali haya**		
M0.7	Diet	MSG	With the next questions, I would like to ask about the foods that you eat. As a reminder you can press the * key for any question that you need to have repeated.<br>**Katika maswali yanayofuata, ningependa kukuuliza kuhusu vyakula unavyokula. Kama kukumbusha unaweza kubonyeza kitufe cha nyota kwa swali lolote unalotaka lirudiwe.**		
Q11 screen	Diet	MC	Do you eat any fruit in a typical week? Press 1 if Yes, Press 3 if no<br>**Kwa kawaida unakula tunda lolote katika wiki? Kama NDIYO bonyeza 1 kama HAPANA bonyeza 3**	1 = YES<br>3 = NO<br>0 = REFUSED<br>OTHER = (Error_0_1)	If 3 OR 0 GO TO 13screen
Q11	Diet	MC	In a typical week, on how many days do you eat fruit? Please enter the number of days now.<br>**Katika wiki, ni siku ngapi unakula matunda? Tafadhari sasa ingiza idadi ya siku unazokula matunda.**	MC, RANGE (1-7)<br>0 = REFUSED<br>OTHER = (Error_0_1)	IF 1-7 GO TO Q12<br>IF 0, GO TO Q13screen
Q12	Diet	MC	A serving of fruit is a medium sized mango, banana or orange or apple or half a cup of cooked or chopped fruit. How many servings of fruit do you eat on one of those days? Please enter the number of servings now.<br>**Mgao wa tunda ni sawa na kiwango cha embe moja au ndizi moja au chungwa moja au nanasi robo au appo moja. Ni migao mingapi ya matunda unakula katika siku hizo? Tafadhali ingiza namba ya migao.**	MC, RANGE (1– 9)<br>0 = REFUSED<br>OTHER = (Error_0_1)	
Q13screen	Diet	MC	Do you eat any vegetables in a typical week? Press 1 if Yes, Press 3 if no<br>**Kwa kawaida unakula mboga za majani katika wiki? Kama NDIYO bonyeza 1, kama HAPANA bonyeza 3**	1 = YES<br>3 = NO<br>0 = REFUSED<br>OTHER = (Error_0_1)	If 3 OR 0 GO TO M0.8
Q13	Diet	MC	In a typical week, on how many days do you eat vegetables? Please enter the number of days now.<br>**Kwa kawaida katika wiki, ni siku ngapi unakula mboga za majani? Tafadhali sasa ingiza namba ya siku unazokula mboga za majani.**	MC, RANGE (1-7)<br>0 = REFUSED<br>OTHER = (Error_0_1)	IF 1-7 GO TO Q14<br>IF 0, GO TO M0.8

Q14	Diet	MC	One serving of vegetables is equivalent to a full cup of green vegetables or salads or half a cup of vegetables either cooked or chopped. How many cupsof vegetables do you eat in day?? Please enter the number of cups now.<br>**Mgao wa mboga za majani ni sawa na kibakuli kidogo kilichojaa mboga za majani zilizokatwakatwa au nusu kibakuli cha mboga zilizopikwa (mfano , mchicha, sukumawiki, spinachi , kisamvu, matembele) Je ni vibakuli vingapi vya mboga za majani unakula kwa siku? Tafadhali sasa ingiza idadi ya bakuli za mboga za majani unazokula.**	MC, RANGE (1-9)<br>0 = REFUSED<br>OTHER = (Error_0_1)	
M0.8	Diet	MSG	With the next questions, I would like to ask about the salt and types of seasonings you eat. As a reminder you can press the * key for any question that you need to have repeated and you can press zero to skip any question you prefer not to answer.<br>**Kwa maswali yanayofuata,ningependa kukuuliza kuhusu chumvi na aina ya viungo vyenye chumvi unazokula.**<br>**Kama kukumbusha unaweza kubonyeza NYOTA kwa swali unalotaka lirudiwe, na unaweza kubonyeza sifuri kuruka swali lolote usilopenda kulijibu.**		
15	Diet	MC	When cooking or preparing food in your household, how often is salt, seasoned salt or salty sauce added in the food? If ALL THE TIME, press 1. If SOMETIMES, press 2. If RARELY, press 3. If NEVER, press 4. If you DON'T KNOW, press 7.<br>**Wakati unapika au kuandaa chakula kwenye kaya, ni mara ngapi chumvi, viungo vyenye asili ya chumvi au sosi ya chumvi unaongeza kwenye chakula? Kama KILA WAKATI, bonyeza 1, kama WAKATI MWINGINE bonyeza 2, kama kwa NADRA, bonyeza 3, kama hakuna kabisa bonyeza 4, KAMA HUJUI, bonyeza 7**	1 = ALWAYS<br>2 = SOMETIMES<br>3 = RARELY<br>4 = NEVER<br>7= DON'T KNOW<br>0 = REFUSED<br>OTHER = (Error_0_1)	
16	Diet	MC	After cooking, how many times do you add salt, salty sauce or seasoned salt in your food when eating? If ALL THE TIME, press 1. If SOMETIMES, press 2. If RARELY, press 3. If NEVER, press 4.<br>**BAADA YA KUPIKA, UKIWA UNAKULA, ni mara ngapi hua unaongeza chumvi, sosi yenye chumvi, viungo vyenye chumvi? Kama KILA WAKATI, bonyeza 1, kama WAKATI MWINGINE bonyeza 2, kama KWA NADRA, bonyeza 3, kama HAKUNA KABISA bonyeza 4,**	1 = ALWAYS<br>2 = SOMETIMES<br>3 = RARELY<br>4 = NEVER<br>0 = REFUSED<br>OTHER = (Error_0_1)	
17	Diet	MC	How often do you eat salty foods such as chutney, salty foods served in fast foods, cheese or processed meat? If ALL THE TIME, press 1. If SOMETIMES, press 2. If RARELY, press 3. If NEVER, press 4.<br>**Ni mara ngapi huwa unakula chakula chenye chumvi nyingi kama vile chachandu, vyakula vilivyookwa kwa chumvi vinavyouzwa kwenye migahawa ya haraka, Jibini au nyama za kwenye makopo**	1 = ALWAYS<br>2 = SOMETIMES<br>3 = RARELY<br>4 = NEVER<br>0 = REFUSED<br>OTHER = (Error_0_1)	

			**kama soseji? kama KILA WAKATI bonzeza 1, kama WAKATI MWINGINE bonyeza 2, kama KWA NADRA bonyeza 3, kama HAKUNA KABISA bonyeza 4.**		
18	Diet	MC	Do you currently do anything daily to limit your salt intake? If YES, press 1. If NO, press 3.<br>**Kwa sasa unafanya chochote kuweka kikomo cha chumvi unayotumia? Kama NDIO, bonyeza 1, kama HAPANA ,bonyeza 3**	1 = YES<br>3 = NO<br>0 = REFUSED<br>OTHER = (Error_0_1)	
M0.9	Diet	MSG	Thank you for answering these questions about the food that you eat<br>**Asante kwa kujibu maswali haya yanayohusu chakula unachokula**		
M1.0	BP & Diab.	MSG	I am now going to ask you questions about some medical conditions and any current medications. As a reminder you can press the * key for any question that you need to have repeated and you can press zero to skip any question you prefer not to answer<br>**Sasa nitaenda kukuuliza maswali juu ya hali yako ya kiafya na kama kwa sasa una matibabu yeyote. Kama kukumbusha unaweza kubonyeza nyota kwa swali lolote ambalo ungetaka lirudiwe na unaweza kubonyeza sifuri kuruka swali lolote ambalo usingependa kulijibu.**		
Q19 Scre en	BP & Diab	MC	Have you ever had your blood pressure measured by a doctor or other health worker? If Yes, Press 1. If no Press 3<br>**Umeshawahi kupimwa shinikizo la damu na mganga/daktari au mhudumu wa afya? Kama Ndiyo bonyeza 1.Kama Hapana bonyeza 3**	1 = YES<br>3 = NO<br>0 = REFUSED<br>OTHER = (Error_0_1)	If 3 or 0, GO to Q21screen
Q19	BP & Diab	MC	Have you ever been told by a doctor or other health worker that you have raised blood pressure or hypertension? If YES, press 1. If NO, press 3.<br>**Umeshawahi kuambiwa na Daktari/mganga au mhudumu wa afya kama una presha/shinikizo la damu? Kama NDIO, bonyeza 1, kama HAPANA bonyeza 3.**	1 = YES<br>3 = NO<br>0 = REFUSED<br>OTHER = (Error_0_1)	IF 3 OR 0, GO TO Q21screen
Q20	BP & Diab	MC	In the last two weeks, have you taken any drugs or medication for raised or high blood pressure that was prescribed to you by a doctor or health worker? If YES, press 1. If NO, press 3.<br>**Katika wiki mbili zilizopita, umechukua dawa au matibabu yoyote kwa ajili ya presha ya kupanda ambazo uliandikiwa na daktari au muhudumu wa afya? Kama NDIO bonyeza 1, kama HAPANA , bonyeza 3**	1 = YES<br>3 = NO<br>0 = REFUSED<br>OTHER = (Error_0_1)	
Q21 Scre en	BP & Diab	MC	Have you ever had your blood sugar measured by a doctor or other health worker? If Yes press 1,If No press 3<br>**Umeshawahi kupimwa sukari na Daktari/mganga au mhudumu wa afya? Kama Ndiyo bonyeza 1, Kama Hapana bonyeza 3**	1 = YES<br>3 = NO<br>0 = REFUSED<br>OTHER = (Error_0_1)	If 3 or 0, GO to M1.1

Q21	BP & Diab	MC	Have you EVER been told by a doctor or health worker that you have raised or high blood sugar or that you have diabetes? If YES, press 1. If NO, press 3.<br>**Ulishawahi kuambiwa na daktari au muhudumu wa afya kwamba una ongezeko la sukari au kisukari? Kama NDIO, bonyeza 1 kama HAPANA bonyeza 3.**	1 = YES<br>3 = NO<br>0 = REFUSED<br>OTHER = (Error_0_1)	IF 3 OR 0, GO TO M1.1
Q22	BP & Diab	MC	In the last two weeks, have you taken any drugs or medications, such as insulin, for diabetes that was prescribed to you by a doctor or health worker? If YES, press 1. If NO, press 3.<br>**Katika wiki mbili zilizopita, umekwisha tumia dawa au matibabu yoyote kwa mfano dawa ya kisukari, kwa ajili ya kisukari ambayo uliandikiwa na daktari au muhudumu wa afya? Kama NDIO, bonyeza 1, kama HAPANA, bonyeza 3**	1 = YES<br>3 = NO<br>0 = REFUSED<br>OTHER = (Error_0_1)	
M1.1	BP & Diab	MSG	Thank you for answering these questions<br>**Asante kwa kujibu maswali haya**		
M1.2	IPAQ	MSG	I am going to ask you about the time you spend being physically active in a typical week. These include activities that you do at work, that you do at home, and that you do in your spare time for recreation or exercise and for at least **ten** minutes. I will ask you about **vigorous** physical activities first and then **moderate** physical activities. As a reminder, you can press the * key for any question that you need repeated<br>**Nitaenda kukuuliza kuhusu muda unaotumia kufanya mazoezi, Mfano hasa katika wiki. Hivi vinajumlisha shughuli/ kazi unazofanya kazini, unazofanya nyumbani, na unazofanya katika muda wako wa ziada. kwa angalau dakika kumi. Nitakuuliza kuhusu kazi /shughuli za kutumia nguvu kisha kazi/shughuli za kawaida za mwili. Kama kukumbusha, unaweza kubonyeza nyota kwa swali lolote unalotaka lirudiwe**		
Q23_Screen	IPAQ	MC	In a typical week, do you do any vigorous physical activity? Vigorous activities means that you breathe much harder than normal for at least ten minutes; like running, fast bicycling, carrying heavy loads of 20 kg or more. If Yes, Press 1. If No, press 3.<br>**Kwa kawaida katika wiki unafanya shughuli/ kazi za mwili za kutumia nguvu? Shughuli/ kazi za mwili za nguvu maana yake ni kwamba unapumua ZAIDI ya kawaida kwa angalau dakika kumi: mfano kukimbia, kuendesha baiskeli haraka, kubeba mzigo mzito wa kilo 20 au zaidi. Kama NDIYO bonyeza 1 na kama HAPANA bonyeza 3**	1 = YES<br>3 = NO<br>0 = REFUSED<br>OTHER = (Error_0_1)	If 3 OR 0, GO TO M1.3
Q23	IPAQ	MC	In a typical week, on how many days do you do **vigorous** physical activities? Please enter the number of days now.<br>**Kwa kawaida katika wiki, ni kwa siku ngapi hasa unafanya kazi/shughuli za kutumia nguvu? Tafadhali ingiza idadi ya siku**	MC, RANGE (1-7)<br>0 = REFUSED<br>OTHER = (Error_0_1)	IF 1-7 GO TO Q24<br>IF 0, GO TO M1.3

Q24	IPAQ	NUM	How many hours do you usually spend doing **vigorous** physical activities on one of those days? If you do 1 hour or less than 1 hour, press 1; if you do about 2 hours, press 2; if you do about 3 hours, press 3 and so on.<br>**Ni masaa mangapi hua unatumia kufanya kazi /shughuli za kutumia nguvu siku moja kati ya siku hizo? Kama unafanya kwa saa moja au chini ya saa, bonyeza 1; kama unafanya kama masaa mawili, bonyeza 2; kama unafanya kwa masaa 3 au zaidi , bonyeza 3 na kuendelea**	MC,RANGE (1-9)<br>0 = REFUSED<br>OTHER = (Error_0_1)	
M1.3	IPAQ	MSG	Now think about activities which take **MODERATE** physical effort that you do in a typical week. Moderate physical activities make you breathe a little harder than normal and may include brisk walking, dancing, digging or housework. Do not include walking. Again, think about only those physical activities that you did for at least 10 minutes at a time.<br>**Sasa fikiria kuhusu shughuli zinazochukua kiwango cha nguvu cha wastani ambazo unafanya hasa kwa mfano katika wiki. Shughuli za mwili za kiwango cha wastani zinakufanya upumue kwa kiwango cha wastani kuliko kupumua kwa kawaida. Mfano ukiwa unatembea kwa haraka, kucheza muziki, kulima, kazi za nyumbani. Usijumuishe kutembea kwa kawaida. Fikiria tena shughuli/kazi ambazo ulizifanya kwa angalau dakika kumi mfululizo.**		
Q25_screen	IPAQ	MC	In a typical week, do you do any moderate physical activity? If Yes, Press 1. If No, press 3.<br>**Kwa kawaida katika wiki unafanya shughuli/kazi za mwili za kiwango cha wastani? Kama NDIYO bonyeza 1, kama HAPANA bonyeza 3.**	1 = YES<br>3 = NO<br>0 = REFUSED<br>OTHER = (Error_0_1)	IF 3 OR 0, go to M1.4
Q25	IPAQ	MC	In a typical week, on how many days do you do **moderate** physical activities? Please enter the number of days now.<br>**Kwa kawaida katika wiki, ni siku ngapi unafanya shughuli za wastani za mwili? Tafadhali ingiza idadi ya siku.**	MC RANGE (1-7)<br>0 = REFUSED<br>OTHER = (Error_0_1)	IF 1-7 GO TO Q26<br>IF 0, GO TO M1.4
Q26	IPAQ	MC	How many hours do you usually spend doing moderate physical activities in a day? If you do 1 hour or less than 1 hour, press 1; if you do about 2 hours, press 2; if for 3 hours, press 3 and so on.<br>**Ni masaa mangapi hua unatumia kufanya shughuli za wastani za mwili kwa siku? Kama unafanya kwa saa 1 au chini ya saa 1, bonyeza 1; kama unafanya kwa masaa 2, bonyeza 2; kama kwa masaa 3 au zaidi, bonyeza 3 na kuendelea**	MC,RANGE (1-9)<br>0 = REFUSED<br>OTHER = (Error_0_1)	
M1.4	IPAQ	MSG	Now think about the time you spent walking in a typical week. This includes at work and at home, walking to travel from place to place, and any other walking that you might do solely for recreation, sport, exercise, or leisure.<br>**Sasa fikiria muda unaoutumia kutembea mfano hasa kwa wiki/juma. Hii inajumuisha katika kazi za nyumbani, kutembea kutoka sehemu**		

			**moja kwenda sehemu nyingine au kutembea kwa aina yoyote ile , ambako ungefanya kama burudani (mfano kucheza ngoma, kucheza mziki au mazoezi)**		
Q27	IPAQ	MC	In a typical week, on how many days do you walk for at least 10 minutes at a time? Please enter the number of days now. For this question, press 9 if you do not want to answer.<br>**Kwa kawaida katika wiki, ni siku ngapi unatembea angalau kwa dakika 10? Tafadhali ingiza namba ya siku. Kwa swali hili bonyeza 9 kama hutaki kujibu**	MC, RANGE (0-7)<br>9 = REFUSED<br>OTHER = (Error_0_1)	IF 1-7 GO TO Q28<br>IF 9, GO TO M1.5
Q28	IPAQ	NUM	How many hours do you usually spend walking on one of those days? If you do 1 hour or less than 1 hour, press 1; if you do about 2 hours, press 2; if for 3 hours, press 3 and so on.<br>**Ni masaa mangapi huwa Unatumia kutembea katika siku hizo? Kama unafanya kwa saa 1 au chini ya saa, bonyeza 1, kama unafanya masaa 2; bonyeza 2, kama masaa 3 au zaidi bonyeza 3 na kuendelea**	MC,RANGE (1-9)<br>0 = REFUSED<br>OTHER = (Error_0_1)	
M1.5	IPAQ	MSG	Thank you for answering questions about physical activity.<br>**Asante kwa kujibu maswali yanayohusu shughuli za mwili**		
M1.6	EXIT	MSG	INSERT APPROPRIATE EXIT MESSAGE		

INTRO MESSAGES			
**Microtrial**	**SN**	**Description**	**Text**
5	T5A_Intro	Full IVR Survey	This interview will take no more than 20 minutes of your time. Any information you share will be kept confidential and private. To answer each question, I will ask you to press a number - for example, 1 for YES, and 3 for NO, or to answer a question with a number - like 12 or 22. You can hear a question again at any time by pressing the STAR button - located to the left of the ZERO at the bottom of your phone. As a token of appreciation, we will send you 5000 Shillings of airtime for completing the survey<br>**Mahojiano haya yatachukua muda wako wa dakika 20. Taarifa yoyote utakayo itoa itatunzwa kwa usiri. Kujibu maswali nitakuomba kubonyeza namba kwa mfano, utabonyeza namba 1 kwa NDIYO au namba 3 KWA HAPANA. Na kujibu maswali kwa kubonyeza namba mfano 12 au 22. Unaweza kusikiliza swali tena muda wowote kwa kubonyeza kitufe cha NYOTA kilichopo upande wa kushoto baada ya kitufe cha SIFURI katika simu yako. Kama namna ya kukushukuru tutakutumia kiasi cha muda wa hewani cha shilingi 5000 kwa kumaliza kujibu dodoso.**
5	T5B_Intro	Human Intro, then IVR	I will now need to ask you some questions to confirm if you are eligible for the survey. You can hear a question again at any time by pressing the STAR button - located to the left of the ZERO at the bottom of your phone. If there is a question that you do not want to answer, you can skip it by pressing ZERO<br><br>**Sasa ningependa kukuuliuza maswali ili kuhakiki kama unastahili kushiriki kwenye utafiti huu.**<br>**Unaweza kusikiliza swali tena muda wowote kwa kubonyeza kitufe cha NYOTA kilichopo upande wa kushoto baada ya kitufe cha SIFURI katika simu yako. Kama kuna swali lolote ambalo usingependa kujibu, unaweza kuruka kwa kubonyeza sifuri.**
7	T7A_Intro	IVR Survey Second	Hello, thank you for taking the survey last week from Ifakara Health Institute. This is the follow up survey they informed you about. This interview will take no more than 20 minutes of your time. Any information you share will be kept confidential and private. To answer each question, I will ask you to press a number - for example, 1 for YES, and 3 for NO, or to answer a question with a number - like 12 or 22. You can hear a question again at any time by pressing the STAR button - located to the left of the ZERO at the bottom of your phone. As a token of appreciation, today we will send you 5000 Shillings of airtime for completing this survey.<br>**Habari, asante kwa kushiriki katika utafiti wiki iliyopita kutoka taasisi ya afya Ifakara.Huu ni utafiti ambao walikwambia wiki iliyopita. Mahojiano haya yatahukua muda wako wa dakika 20. Taarifa yoyote utakayo itoa itatunzwa kwa usiri. Kujibu maswali nitakuomba kubonyeza namba kwa mfano utabonyeza namba 1 kwa NDIYO au namba 3 KWA HAPANA. Na kujibu maswali kwa kubonyeza namba mfano 12 au 22. Unaweza kusikiliza swali tena muda wowote kwa kubonyeza kitufe cha NYOTA kilichopo upande wa kushoto baada ya kitufe cha SIFURI katika simu yako. Kama namna ya kukushukuru tutakutumia kiasi cha muda wa hewani cha shilingi 5000 kwa kumaliza kujibu dodoso.**
7	T7B_Intro	IVR Survey First	This interview will take no more than 20 minutes of your time. Any information you share will be kept confidential and private. To answer each question, I will ask you to press a number - for example, 1 for YES, and 3 for NO, or to answer a question with a number - like 12 or 22. You can hear a question again at any time by pressing the STAR button - located to the left of the ZERO at the bottom of your phone. As a token of appreciation, today we will send you 5000 Shillings of airtime for completing this survey. We will call you again in one week to take another survey where you will receive an additional 5000 shillings of airtime<br>**Mahojiano haya yatachukua muda wako wa dakika 20. Taarifa yoyote utakayo itoa itatunzwa kwa usiri. Kujibu**

			**maswali nitakuomba kubonyeza namba kwa mfano, utabonyeza namba 1 kwa NDIYO au namba 3 KWA HAPANA. Na kujibu maswali kwa kubonyeza namba mfano 12 au 22. Unaweza kusikiliza swali tena muda wowote kwa kubonyeza kitufe cha NYOTA kilichopo upande wa kushoto baada ya kitufe cha SIFURI katika simu yako. Kama namna ya kukushukuru tutakutumia kiasi cha muda wa hewani cha shilingi 5000 kwa kumaliza kujibu dodoso.Tutakupigia tena baada ya wiki moja kwa ajili ya kushiriki katika utafiti huu tena na utapewa tena muda wa hewani wenye thamani ya shilingi 5000.**

**EXIT MESSAGES**			
**Microtrial**	SN	Description	Text
5	T5A_Exit	Full IVR Survey	The survey is now finished. Thank you for your time. As a token of appreciation, we will send you airtime worth 5000 Shillings<br>**Tumemaliza mahojiano. Asante kwa muda wako. Kama namna ya kukushukuru tutakutumia muda wa hewani/maongezi wenye thamani ya shilingi za Kitanzania 5000.**
5	T5B_Exit	Human Intro, then IVR	The survey is now finished. Thank you for your time. As a token of appreciation, we will send you airtime worth 5000 Shillings<br>**Tumemaliza mahojiano. Asante kwa muda wako. Kama namna ya kukushukuru tutakutumia muda wa hewani/maongezi wenye thamani ya shilingi za Kitanzania 5000.**
7	T7A_Exit	IVR Survey Second	The survey is now finished. Thank you for your time. As a token of appreciation, we will send you airtime worth 5000 Shillings<br>**Tumemaliza mahojiano. Asante kwa muda wako. Kama namna ya kukushukuru tutakutumia muda wa hewani/maongezi wenye thamani ya shilingi za Kitanzania 5000.**
7	T7B_Exit	IVR Survey First	The survey is now finished. Thank you for your time. As a token of appreciation, we will send you airtime worth 5000 Shillings. We will call you back in one week to take another health survey!!<br>**Tumemaliza mahojiano. Asante kwa muda wako. Kama namna ya kukushukuru tutakutumia muda wa hewani/maongezi wenye thamani ya shilingi za Kitanzania 5000. Tutakupigia tena baada ya wiki moja ili kushiriki katika utafiti huu tena.**
